# c-Myc targeted regulators of cell metabolism in a transgenic mouse model of papillary lung adenocarcinoma

**DOI:** 10.18632/oncotarget.11804

**Published:** 2016-09-01

**Authors:** Yari Ciribilli, Prashant Singh, Alberto Inga, Jürgen Borlak

**Affiliations:** ^1^ Centre for Integrative Biology (CIBIO), University of Trento, 38123 Povo, Italy; ^2^ Centre for Pharmacology and Toxicology, Hannover Medical School, 30625 Hannover, Germany

**Keywords:** c-Myc transgenic mouse model of papillary lung adenocarcinomas, whole genome transcriptome profiling, regulatory gene networks, c-Myc targeted regulators of cellular growth, c-Myc DNA binding activity

## Abstract

c-Myc's role in pulmonary cancer metabolism is uncertain. We therefore investigated c-Myc activity in papillary lung adenocarcinomas (PLAC). Genomics revealed 90 significantly regulated genes (> 3-fold) coding for cell growth, DNA metabolism, RNA processing and ribosomal biogenesis and bioinformatics defined c-Myc binding sites (TFBS) at > 95% of up-regulated genes. EMSA assays at 33 novel TFBS evidenced DNA binding activity and ChIP-seq data retrieved from public repositories confirmed these to be c-Myc bound. Dual-luciferase gene reporter assays developed for RNA-Terminal-Phosphate-Cyclase-Like-1(RCL1), Ribosomal-Protein-SA(RPSA), Nucleophosmin/Nucleoplasmin-3(NPM3) and Hexokinase-1(HK1) confirmed c-Myc functional relevance and ChIP assays with HEK293T cells over-expressing ectopic c-Myc demonstrated enriched c-Myc occupancy at predicted TFBS for RCL1, NPM3, HK1 and RPSA. Note, c-Myc recruitment on chromatin was comparable to the positive controls CCND2 and CDK4. Computational analyses defined master regulators (MR), i.e. heterogeneous nuclear ribonucleoprotein A1, nucleolin, the apurinic/apyrimidinic endonuclease 1, triosephosphate-isomerase 1, folate transporter (SLC19A1) and nucleophosmin to influence activity of up to 90% of PLAC-regulated genes. Their expression was induced by 3-, 3-, 6-, 3-, 11- and 7-fold, respectively. STRING analysis confirmed protein-protein-interactions of regulated genes and Western immunoblotting of fatty acid synthase, serine hydroxyl-methyltransferase 1, arginine 1 and hexokinase 2 showed tumor specific induction. Published knock down studies confirmed these proteins to induce apoptosis by disrupting neoplastic lipogenesis, by endorsing uracil accumulation and by suppressing arginine metabolism and glucose-derived ribonucleotide biosynthesis. Finally, translational research demonstrated high expression of MR and of 47 PLAC up-regulated genes to be associated with poor survival in lung adenocarcinoma patients (HR 3.2 *p* < 0.001) thus, providing a rationale for molecular targeted therapies in PLACs.

## INTRODUCTION

Lung cancer is the leading cause of cancer-related death in the world and there is conclusive evidence for tobacco smoke to be the most important risk factor for disease onset and progression. Recent estimates suggest more than 5 million people to die each year because of tobacco use. Thus, the “tobacco epidemic” has become the biggest challenges to public health with possibly more than 1 billion people around the world being active smokers [1, 2].

Malignancies of the lung are classified as small cell (SCLC) and non-small cell lung cancer (NSCLC); the latter accounts for > 80% of all lung cancers and is typically divided into adeno-, squamous and large cell carcinomas. Noteworthy, adenocarcinomas are most common among NSCLCs and continue to rise [3].

There is a plethora of information on the harmful effects of tobacco smoke to cause lung cancer; however, molecular causes in non-smokers are poorly understood. Importantly, there is evidence for the c-Myc protein to play a central role in various malignancies [4] with *c-Myc* over-expression being also observed in lung carcinomas [5–7]. The c-Myc protein functions as a helix-loop-helix transcription factor and is a key regulator of cell proliferation and cell fate decision; its activity is tightly controlled by mitogens [8]. In tumor cells *c-Myc* expression is usually increased and no longer dependent on external signals. The c-Myc protein recognizes E-box sequences at targeted promoters [9, 10]. However, its ability to transactivate or to repress tumor-associated gene expression is dependent on forming heterodimeric protein complexes with MAX and other transcription factors of the same family. Identifying c-Myc target genes and their downstream effectors is a crucial step towards an understanding of c-Myc-induced carcinogenesis. Although a large number of genes have been shown to be bound by c-Myc and to change their expression accordingly [11], a cell metabolism network in papillary lung adenocarcinoma (PLACs) has not been investigated as yet. We therefore investigated the role of c-Myc in a transgenic disease model. By use of the surfactant protein C promoter, targeted expression of c-Myc to alveolar epithelium was achieved [12]. This led to malignant transformation of respiratory epithelium causing growth of invasive PLACs. The subsequently performed genomic study on histologically well characterized c-Myc transgenic tumors identified 463 differentially expressed genes. Initially, we reported c-Myc networks of cell cycle and apoptosis regulated genes [12]. In the present study we focus on an identification of cell metabolism genes involved in cell growth and employed diverse experimental and computational biology strategies to search for regulatory gene networks. Subsequent validation of c-Myc DNA binding activity was achieved by electrophoretic mobility band shift (EMSA), RT-PCR, Western blotting, gene reporter and ChIP assays. Further evidence for the newly identified target genes stems from the ENCODE ChIP sequence database which provided confirmation for 94% of the 87 up-regulated genes to be c-Myc targets as evaluated in 2 murine as well as 7 different human cell lines including human embryonic stem cells. Moreover, translational research identified high expression of 50 PLAC regulated genes (47 up- and 3 down-regulated) as significantly associated with survival of lung cancer patients. Overall, new biologically relevant c-Myc down-stream effectors were identified that will help defining c-Myc's oncogenic activity in lung cancer metabolism and will aid the development of molecularly targeted therapies.

## RESULTS

In our first report details of the c-Myc transgenic disease model was given and this included an in-depth description of the observed lung cancer pathology [12]. Specifically, targeted expression of c-Myc to the lung was achieved by use of a gene construct that consisted of the surfactant C promoter, the first exon and intron of the non-coding alpha 1 antitrypsin gene fused to the c-*Myc* proto-oncogene and the SV40 Poly A dependent transcription termination signal.

**Figure 1:**
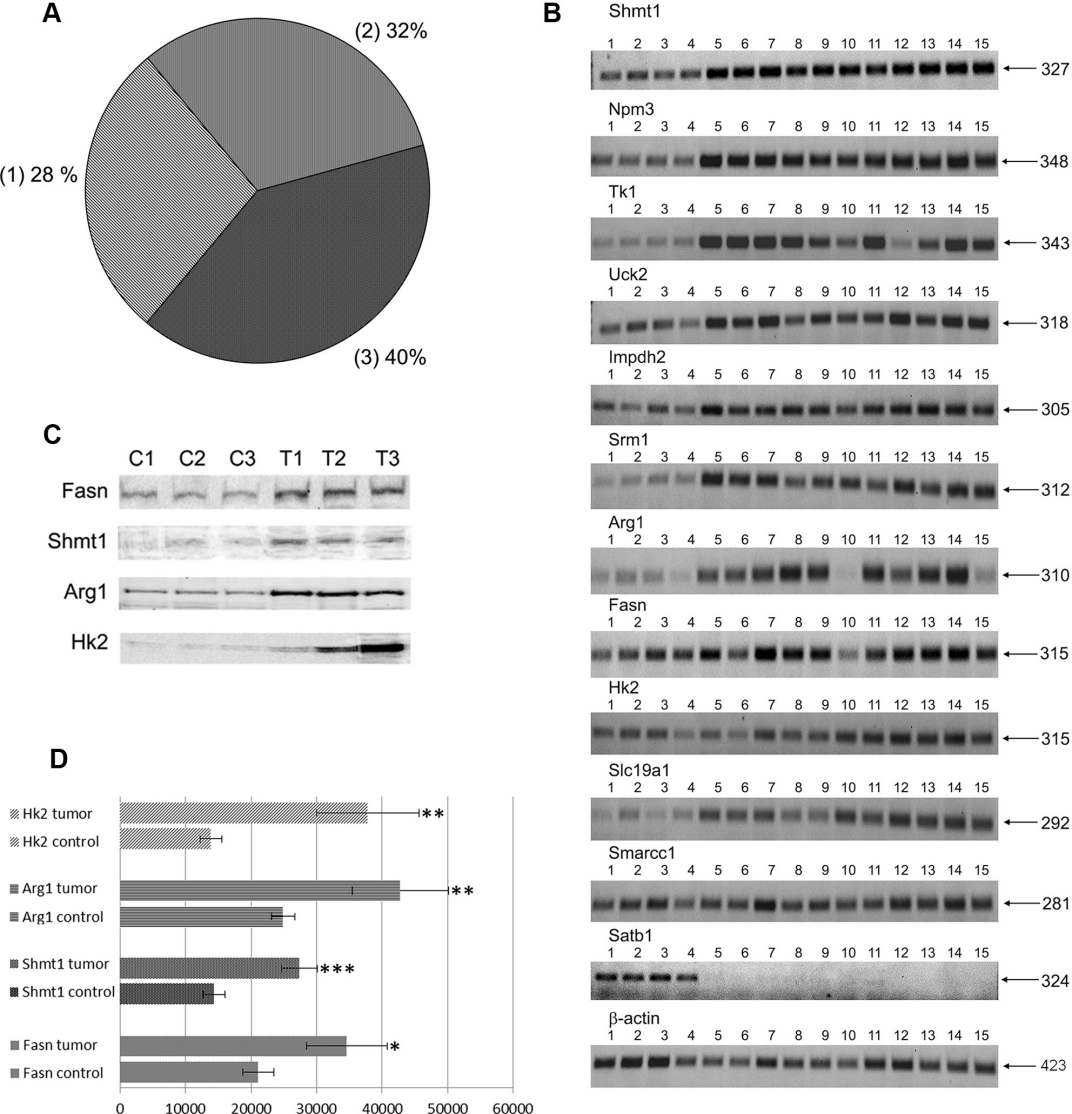
(**A**) Distribution of c-*Myc* regulated genes in PLACs of transgenic mice. 1 - known c-*Myc*-targets and their relatives 2 - known c-*Myc* -responsive genes and relatives 3 - new c-*Myc* -responsive genes. (**B**) RT-PCRs for selected genes: lanes 1–4: control lung; lanes 5–7: pools of small-sized tumors; lanes 8–12: middle-sized tumors; lanes 13–15: large-sized tumors. (**C**) Western blot analysis for selected genes: lanes C1-C-3 - control non-transgenic lung; lanes T1-T3 - lung adenocarcinomas of SPC/c-*Myc* transgenic mice. (**D**) Densitometric scans of Western blots; *p < 0.05, **p < 0.02; ***p < 0.01.

Solid lung tumors classified as papillary lung adenocarcinomas were studied by whole genome transcript profiling. Data from small-, middle- and large-sized tumors were considered separately. As shown in Table 1, the significantly regulated genes code for cell metabolism, DNA and RNA synthesis, ribosomal biogenesis, protein synthesis and transport. The promoter sequences of regulated genes were interrogated for E-box motives and the results were compared with public available datasets where lists of c-*Myc* target genes were reported [13–15]. As summarized in Table 1 and Figure 1 panel A about one third of the genes involved in metabolism and growth proved to be known c-Myc targets (designated as “T” in Table 1) or their relatives (“rT”), i.e. they belong to gene families whose members have been shown to be directly bound by c-Myc. Amongst them were genes coding for thymidilate synthase (TS) and thymidine kinase (TK), both of which are well-established paradigms in anticancer treatment regimens. Indeed, 5-Fluoruracil, i.e. an analogue of uracil inhibits thymidilate synthase; it is in clinical use for decades. Additional examples include spermidine synthase, nucleolin, nucleophosmin, cytoplasmic polyA binding protein 4, various ribosomal proteins, eukaryotic translation initiation factor 3 and chaperonin subunit 5, all of which are critically involved in cancer biology and are suitable targets in anticancer therapy. Furthermore, 20% of regulated genes are known to be c-*Myc*-responsive genes (“R”) or their relatives (“rR”) and include hexokinase 2, glucose phosphatase isomerase 1 and inosine-5′-monophosphate dehydrogenase 2. Note, the latter enzyme is rate limiting in guanine nucleotide biosynthesis. We also observed *Gart*, coding for a tri-functional enzyme to be significantly induced; the enzyme influences de novo purine biosynthesis. Other examples include helicase, RNA polymerase 1–3, replication factor C (activator1) 4 and nucleolar protein 56, e.g. a component of small nucleolar ribonucleoprotein particles that either act as facilitator of malignancies or are involved in aberrant mRNA translation and therefore serve as potential target and new methodologies for cancer treatment (Table 1). Given that induction of these genes had been reported for diverse tumor cell lines in response to c-Myc activation, they can be considered as candidate genes in c-Myc-induced carcinogenesis. Apart from the discovery of known c-Myc targets this transgenic disease model allowed for an identification of novel c-Myc-responsive genes in lung cancer and included arginase1, ribonucleotide reductases M1 and M2, uridine-cytidine kinase 2, meiotic recombination 11a, nucleoplasmi 3, nucleolar proteins and importin 4 whose altered expression can now be linked to adenocarcinomas of the lung. Remarkably, several genes involved in the regulation of transcription through chromatin remodeling were significantly regulated and involved up-regulated helicase, *Smarcc1*, histone H1fX, and laminB1, as well as repressed histone1, H2bc and negative regulators of histone acetylation such as *Satb1* and *Anp32a*. Additionally, in PLACs a significant increase of the SNRP transcript was observed; the gene codes for a dsRNA binding protein and is involved in translation, RNA editing and RNA stability. These findings suggest that chromatin remodeling and posttranscriptional RNA editing may be additional mechanisms by which c-Myc augments expression of genes.

**Table 1:** Gene expression signature in c-Myc-induced lung papillary adenocarcinoma: differentially expressed genes* involved in stimulation of cell proliferation and growth

ACC	Gene Symbol	Gene Title		*N*	Fold Change			% Increase/Decrease**			*p*-value in *T*-test compared to control lung		
					size of tumors			size of tumors			size of tumors		
					small	middle	large	small	middle	large	small	middle	large
**Cell growth, metabolism, glycolysis**													
U51805	*Arg1*	arginase 1, liver		**0**	6,1	10,8	14,8	94	100	100	0,01	0,09	0,27
X13135	*Fasn*	fatty acid synthase	**T, R**	**3**	3,1	2,9	3,1	100	100	100	0,00	0,03	0,09
AB033887	*Acsl4*	acyl-CoA synthetase long-chain family member 4	**rR**	**1**	2,7	3,2	3,2	91	88	100	0,09	0,02	0,00
Z67748	*Srm*	spermidine synthase	**T, R**	**3**	4,6	3,4	5,3	100	88	100	0,00	0,15	0,05
AA913994	*Shmt1*	serine hydroxymethyl transferase 1 (soluble)	**T, R**	**3**	7,5	7,3	9,4	100	100	100	0,01	0,04	0,04
AI841389	*Eno1*	enolase 1, alpha non-neuron	**T, R**	**1**	2,3	2,3	3,5	100	100	100	0,00	0,01	0,05
M17516	*Ldha*	lactate dehydrogenase A	**T, R**	**3**	3,4	3,3	3,7	100	100	100	0,01	0,01	0,03
Y11666	*Hk2*	hexokinase 2	**R**	**2**	2,1	3,6	4,8	100	100	100	0,01	0,03	0,02
J05277	*Hk1*	hexokinase 1		**3**	2,8	2,0	3,2	100	75	100	0,01	0,13	0,00
M32599	*Gapgh*	glyceraldehyde-3-phosphate dehydrogenase		**2**	3,0	2,9	3,5	100	100	100	0,01	0,00	0,04
AF058956	*Suclg2*	succinate-Coenzyme A ligase, GDP-forming, beta subunit		**2**	2,2	2,2	3,0	100	94	100	0,00	0,01	0,02
M14220	*Gpi1*	glucose phosphate isomerase 1	**R**	**2**	4,2	2,0	2,7	100	50	33	0,05	0,29	0,36
L31777	*Tpi1*	triosephosphate isomerase 1		**2**	2,2	2,8	3,4	100	100	100	0,01	0,00	0,01
AW123026	*Gnpnat1*	glucosamine-phosphate N-acetyltransferase 1		**1**	2,8	2,7	3,5	100	94	100	0,04	0,04	0,02
AB030316	*Pign*	phosphatidylinositol glycan, class N		**1**	2,8	1,8	3,2	50	50	100	0,03	0,29	0,00
**Nucleotide biosynthesis, DNA metabolism**													
M13352	*Tyms*	thymidylate synthase	**T, R**	**2**	3,7	2,2	2,3	100	63	75	0,00	0,12	0,20
X60980	*Tk1*	thymidine kinase 1	**T, R**	**4**	4,2	2,5	2,9	100	19	33	0,00	0,05	0,22
U20892	*Gart*	phosphoribosylglycinamide formyltransferase	**R**	**1**	3,0	2,6	3,4	100	94	100	0,01	0,06	0,04
M33934	*Impdh2*	inosine 5 ′ -phosphate dehydrogenase 2	**R**	**2**	4,5	3,5	4,5	100	100	100	0,01	0,03	0,02
AI850362	*Uck2*	uridine-cytidine kinase 2		**2**	4,4	2,8	4,5	100	100	100	0,00	0,06	0,02
U60318	*Mre11a*	meiotic recombination 11 homolog A (S. cerevisiae)		**1**	3,6	2,5	3,2	100	50	100	0,02	0,02	0,01
U01915	*Top2a*	topoisomerase (DNA) II alpha		**1**	10,6	4,8	7,2	100	50	33	0,05	0,07	0,27
L32836	*Ahcy*	S-adenosylhomocysteine hydrolase	**R**	**1**	3,1	2,3	3,0	100	88	100	0,00	0,03	0,07
AI841645	*Naa10*	N(alpha)-acetyltransferase 10, NatA catalytic subunit		**3**	3,3	2,7	2,9	100	100	100	0,00	0,03	0,02
X67668	*Hmgb2*	high mobility group box 2		**1**	4,3	2,4	2,7	100	75	75	0,01	0,14	0,24
D90374	*Apex1*	apurinic/apyrimidinic endonuclease 1	**T,R**	**7**	6,6	4,0	5,9	100	100	100	0,00	0,06	0,00
X66323	*Xrcc5*	X-ray repair complementing defective repair in Chinese hamster cells 5		**2**	3,5	3,0	3,3	100	100	100	0,00	0,00	0,02
K02927	*Rrm1*	ribonucleotide reductase M1		**1**	3,8	2,2	3,2	100	25	33	0,00	0,12	0,31
M14223	*Rrm2*	ribonucleotide reductase M2		**1**	5,8	2,7	3,8	100	6	66	0,00	0,06	0,24
AW122092	*Rfc4*	replication factor C (activator 1) 4	**R**	**1**	4,7	3,1	3,6	100	75	100	0,01	0,05	0,03
M38700	*Xrcc6*	X-ray repair complementing defective repair in Chinese hamster cells 6	**T**	**3**	4,1	3,2	4,0	100	94	100	0,00	0,09	0,00
U25691	*Hells*	helicase, lymphoid specific	**R**	**3**	3,3	1,6	3,1	100	75	100	0,00	0,01	0,10
**Chromatin structure**													
U85614	*Smarcc1*	SWI/SNF related, matrix associated, actin dependent regulator of chromatin, subfamily c, member 1		**1**	3,0	2,2	2,6	100	81	100	0,02	0,02	0,00
AI851599	*H1fx*	H1 histone family, member X		**2**	4,8	5,8	6,4	100	8	24	0,03	0,10	0,14
M35153	*Lmnb1*	lamin B1		**3**	3,8	2,1	2,7	100	56	91	0,01	0,07	0,07
**RNA processing, ribosomal biogenesis**													
AI853173	*Polr1d*	polymerase (RNA) I polypeptide D	**rR**	**2**	3,2	2,7	3,0	100	81	100	0,00	0,04	0,00
AI838709	*Strbp*	spermatid perinuclear RNA binding protein		**1**	6,1	7,1	5,3	100	100	100	0,01	0,02	0,02
AW121447	*Nop56*	NOP56 ribonucleoprotein	**R**	**3**	5,4	3,7	5,2	100	81	100	0,01	0,11	0,02
AA656775	*Rrs1*	RRS1 ribosome biogenesis regulator homolog (S. cerevisiae)	**R**	**2**	4,2	2,9	4,6	100	75	100	0,00	0,08	0,03
X07699	*Ncl*	nucleolin	**T, R**	**1**	3,5	2,7	3,5	100	81	100	0,01	0,03	0,02
M33212	*Npm1*	nucleophosmin 1	**T,R**	**2**	3,7	3,1	4,0	100	100	100	0,00	0,02	0,01
U64450	*Npm3*	nucleoplasmin 3		**1**	6,0	5,5	7,4	100	94	100	0,01	0,05	0,04
AI183202	*Hnrnpa1*	heterogeneous nuclear ribonucleoprotein A1	**T, R**	**2**	2,7	2,1	3,0	100	75	100	0,00	0,04	0,02
Z22593	*Fbl*	fibrillarin	**R**	**1**	3,2	2,8	3,6	100	94	100	0,00	0,02	0,01
AF053232	*Nop58*	NOP58 ribonucleoprotein		**2**	7,7	4,8	6,6	100	100	100	0,00	0,04	0,02
AW060597	*Snrpg*	small nuclear ribonucleoprotein polypeptide G	**rT**	**3**	2,7	2,3	3,2	100	81	100	0,02	0,10	0,04
AA684508	*Snord22*	small nucleolar RNA, C/D box 22	**rT**	**2**	4,8	3,1	3,8	100	69	83	0,00	0,03	0,02
AI853113	*nudt21*	nudix (nucleoside diphosphate linked moiety X)-type motif 21	**rT**	**1**	2,9	2,6	3,1	100	88	100	0,01	0,02	0,03
AA656757	*Pabpc4*	poly A binding protein, cytoplasmic 4	**T**	**2**	5,2	4,0	5,8	100	94	100	0,00	0,04	0,03
AI851198	*Gar1*	GAR1 ribonucleoprotein homolog (yeast)		**0**	4,6	3,6	4,9	100	88	100	0,01	0,07	0,02
AI852665	*Mki67ip*	Mki67 (FHA domain) interacting nucleolar phosphoprotein		**1**	5,2	3,3	3,6	100	88	100	0,01	0,11	0,01
AA674812	*Ppan*	peter pan homolog (Drosophila)		**1**	4,0	2,6	3,7	100	75	100	0,01	0,06	0,00
AI852608	*Rcl1*	RNA terminal phosphate cyclase-like 1		**2**	3,6	2,7	4,0	100	88	100	0,02	0,04	0,00
AI845664	*Grwd1*	glutamate-rich WD repeat containing 1		**0**	3,8	2,1	3,2	91	44	100	0,00	0,16	0,02
**Protein synthesis and metabolism**													
AW122030	*Psat1*	phosphoserine aminotransferase 1	**T, R**	**3**	7,2	5,3	5,5	100	100	100	0,02	0,03	0,03
U12403	*Rpl10a*	ribosomal protein L10A	**T, R**	**1**	2,6	2,2	3,2	100	81	100	0,00	0,05	0,03
X51528	*Rpl13a*	ribosomal protein L13a	**T, R**	**2**	6,3	4,3	5,0	100	100	100	0,00	0,02	0,04
X05021	*Rpl27a*	ribosomal protein L27a	**T, R**	**1**	2,9	2,4	3,3	100	75	100	0,00	0,05	0,02
AW060951	*Bzw2*	basic leucine zipper and W2 domains 2	**rT**	**2**	5,4	4,1	6,2	100	81	100	0,00	0,10	0,00
AI839363	*Eif3e*	eukaryotic translation initiation factor 3, subunit E	**T, R**	**1**	4,1	3,2	4,4	100	75	100	0,00	0,09	0,01
AV170770	*Cct5*	chaperonin subunit 5 (epsilon)	**T, R**	**1**	4,1	3,4	3,6	100	75	100	0,00	0,05	0,00
AW122851	*Fkbp11*	FK506 binding protein 11	**rT**	**4**	7,9	7,5	9,1	100	75	100	0,00	0,12	0,05
AI840579	*Srr*	serine racemase		**1**	4,5	3,6	5,0	100	100	100	0,00	0,04	0,02
AW124889	*Aldh18a1*	aldehyde dehydrogenase 18 family, member A1		**2**	3,6	2,0	4,4	100	50	100	0,00	0,04	0,01
AW046590	*Pcbd1*	pterin 4 alpha carbinolamine dehydratase/dimerization cofactor of hepatocyte nuclear factor 1 alpha (TCF1) 1		**1**	5,0	4,3	5,0	100	100	100	0,01	0,01	0,03
AI840436	*Mrps5*	mitochondrial ribosomal protein S5		**2**	2,4	2,2	3,3	100	50	100	0,09	0,01	0,00
X06406	*Rpsa*	ribosomal protein SA		**2**	2,7	2,2	3,1	83	75	100	0,01	0,04	0,01
AW124432	*Mrpl12*	mitochondrial ribosomal protein L12		**2**	4,0	3,5	3,2	100	88	100	0,00	0,07	0,01
AW045418	*Rpl36a*	ribosomal protein L36A-like		**4**	2,6	2,1	3,1	100	75	100	0,00	0,05	0,01
AW120719	*Eif2b1*	eukaryotic translation initiation factor 2B, subunit 1 (alpha)		**2**	4,3	3,2	3,3	100	94	100	0,00	0,03	0,00
**Transport**													
AW125446	*Golm1*	golgi membrane protein 1	**rT**	**3**	4,1	5,1	4,6	100	100	100	0,01	0,00	0,04
AW122428	*Timm10*	translocase of inner mitochondrial membrane 10 homolog (yeast)	**rR**	**3**	5,7	3,3	4,2	100	50	100	0,01	0,14	0,03
AA655369	*Timm8a1*	translocase of inner mitochondrial membrane 8A1	**rR**	**2**	5,8	4,5	5,7	100	100	100	0,00	0,07	0,01
AF043249	*Tomm40*	translocase of outer mitochondrial membrane 40 homolog (yeast)		**1**	2,7	2,3	3,2	100	38	100	0,00	0,00	0,00
AI846308	*Sfxn1*	sideroflexin 1	**R**	**2**	2,9	2,4	3,1	100	88	100	0,00	0,05	0,00
L23755	*Slc19a1*	solute carrier family 19 (sodium/hydrogen exchanger), member 1	**T**	**2**	10,4	6,6	11,1	100	75	100	0,02	0,04	0,03
AI846682	*Slc15a2*	solute carrier family 15 (H+/peptide transporter), member 2		**2**	2,1	2,7	3,6	66	88	100	0,21	0,02	0,03
AI594427	*Slc4a7*	solute carrier family 4, sodium bicarbonate cotransporter, member 7		**2**	2,5	2,3	3,1	100	88	100	0,00	0,04	0,00
AF020195	*Slc4a4*	solute carrier family 4 (anion exchanger), member 4		**1**	2,1	4,2	4,3	91	100	100	0,01	0,09	0,05
U88623	*Aqp4*	aquaporin 4	**rR**	**2**	7,1	5,0	4,9	100	75	66	0,02	0,08	0,25
AI846319	*Rangrf*	RAN guanine nucleotide release factor		**2**	4,2	3,6	5,7	100	50	91	0,00	0,05	0,00
D55720	*Kpna2*	karyopherin (importin) alpha 2	**rT**	**1**	3,2	1,8	1,7	100	56	41	0,03	0,17	0,36
AI847564	*Ipo5*	importin 5		**1**	3,9	2,7	3,8	100	94	100	0,02	0,03	0,01
AW212243	*Ipo4*	importin 4		**2**	3,6	2,8	3,7	100	81	100	0,00	0,01	0,00
X61399	*Mlp (Csrp3)*	MARCKS-like protein		**0**	3,3	2,5	4,6	100	94	100	0,01	0,02	0,03
M60348	*Abcb1b*	ATP-binding cassette, sub-family B (MDR/TAP), member 1B	**rT**	**1**	2,0	1,6	3,2	83	50	100	0,02	0,28	0,00
AF099988	*Stk39*	serine/threonine kinase 39, STE20/SPS1 homolog (yeast)		**1**	3,3	3,3	4,3	100	100	100	0,01	0,01	0,02
**Chromatin remodelling**													
U05252	*Satb1*	special AT-rich sequence binding protein 1		**1**	−5,4	−5,6	–7,0	100	100	100	0,01	0,01	0,00
U73478	*Anp32a*	acidic (leucine-rich) nuclear phosphoprotein 32 family, member A		**3**	–1,7	–3,6	–3,8	66	100	100	0,29	0,00	0,01
X05862	*Hist1h2bc*	histone 1, H2bc	**rT**	**2**	–3,0	−3,0	–4,9	100	100	100	0,00	0,00	0,00

* Criteria for significant expression changes were as follows: a gene should be expressed (“Present” call) in all tumor replicates of one set or in all control non-transgenic lungs for up- and down-regulated genes respectively and have a mean fold change (FC) > 3, *p*-value in *T*-test < 0.05 and 100% “Increase” call in comparative ranking analysis for up regulated genes and FC < - 3, *p*-value in *T*-test < 0.05 and 100% “Decrease” for down regulated genes.

T- known c-Myc-target.

R- known c-Myc responsive gene.

rT- relative of known c-Myc-target.

rR- relative of known c-Myc-responsive gene.

N- number of potential c-Myc binding sites in promotor sequence.

** - % Increase/Decrease - concordance of change calls in the pairwise comparisons (each tumor to each normal lung sample), by which genes are up or down regulated respectively.

An important finding of the microarray study is the lack of qualitative differences in the gene expression profiles of papillary adenocarcinomas of various sizes. However, quantitative differences in the expression of *Tk1*, *Top2a*, *Hmgb2*, *Rrm2*, *Kpna2*, and *H1fx* were observed and were found to be strongly up-regulated in small-sized adenocarcinomas. This may suggest a specific role of these genes at the onset of PLAC development. Other genes, i.e. arginase1, hexokinase2, and anion exchanger *Slc4a4*, were expressed at higher levels (more than 2-fold) in large tumors when compared with small-sized tumors, pointing to their essential function in malignant growth (Table 1).

It is of considerable importance that induction of certain genes in tumors was observed whose expression was absent in non-transgenic controls (marked by a grey background for the gene title in Table 1); some were consistently increased in all 10 tumors studied (marked by grey rows accordingly in Table 1). The uniquely expressed genes, such as arginase1 and serine hydroxymethyl transferase, are interesting “druggable” candidates for molecularly targeted tumor therapies.

### RT-PCR and Western blotting of regulated genes

Twelve genes were selected for validation through reverse transcription-polymerase chain reaction (RT-PCR) to confirm their regulation by an alternative method. The results obtained by both methods agreed well; for example, genes that were up-regulated in tumor cells in the microarrays were consistently over-expressed when measured by RT-PCR (Figure 1 panel B and Table 2). Moreover, the protein expression levels of Fasn, Shmt1, Arg1, and Hk2, that have important functions in cell proliferation and growth, were studied by Western blot analysis and these experiments confirmed their up-regulation in lung adenocarcinomas (see Figure 1 panel C and D).

**Table 2:** Fold changes of gene expression in lung tumors determined by RT-PCR and microarray analysis

Gene	Method	Mean FC		
		size of tumors		
		small	middle	large
***Shtm1***	RT-PCR	3,1	3,1 ± 0,0	3,5 ± 0,3
	Affymetrix	7,5 ± 0,3	7,3 ± 2,0	9,4 ± 1,0
***Npm3***	RT-PCR	4,3 ± 1,4	2,8 ± 0,6	3,0 ± 0,6
	Affymetrix	6,0 ± 0,7	5,5 ± 1,5	7,4 ± 1,1
***Tk1***	RT-PCR	8,3 ± 2,5	4,4 ± 2,2	5,3 ± 1,3
	Affymetrix	4,2 ± 0,1	2,5 ± 0,3	2,9 ± 0,5
***Uck2***	RT-PCR	3,1	1,9 ± 0,3	2,4
	Affymetrix	4,4 ± 0,1	2,8 ± 0,9	4,5 ± 0,4
***Impdh2***	RT-PCR	2,5 ± 1,0	2,3 ± 0,4	2,6 ± 1,4
	Affymetrix	4,5 ± 0,4	3,5 ± 1,3	4,5 ± 0,8
***Srm1***	RT-PCR	5,4	4,4 ± 1,9	4,1
	Affymetrix	4,6 ± 0,2	3,4 ± 1,7	5,3 ± 0,8
***Arg1***	RT-PCR	6,5 ± 1,2	4,0	4,6
	Affymetrix	6,1 ± 0,6	10,8 ± 1,9	14,8 ± 4,7
***Fasn***	RT-PCR	2,5 ± 0,6	1,7 ± 0,6	2,1 ± 0,6
	Affymetrix	3,1 ± 0,1	2,9 ± 0,7	3,1 ± 0,7
***Hk2***	RT-PCR	1,5 ± 0,3	2,1 ± 0,7	2,3 ± 0,5
	Affymetrix	2,1 ± 0,2	3,6 ± 1,4	4,8 ± 0,5
***Slc19a1***	RT-PCR	5,3 ± 1,5	4,5 ± 2,2	5,6 ± 0,8
	Affymetrix	10,4 ± 0,1	6,6 ± 0,5	11,1 ± 0,3
***Smarcc1***	RT-PCR	1,9 ± 0,1	1,5 ± 0,3	1,6 ± 0,2
	Affymetrix	3,0 ± 0,4	2,2 ± 0,1	2,6 ± 0,1
***Satb1***	RT-PCR	A	A	A
	Affymetrix	–5,4 ± 0,2	–5,6 ± 0,4	–7,0 ± 0,7

A - “Absent” - no expression was detected.

RT-PCR image analysis was done with the NIH ImageJ software (https://imagej.nih.gov). Data are given as mean FC and SD relative to control.

### Mapping of c-Myc binding sites in genomic sequences of differentially expressed genes

A bioinformatics strategy to search for c-Myc regulatory gene networks in lung adenocarcinomas of c-Myc transgenic mice was previously published [16]. c-Myc binding sites at gene specific promoter sequences were considered in the range of 1000 bp upstream and 100 bp downstream of the TSS using 9 different PWMs. Specifically, the PWMs recognize E-box motives based on the core consensus sequence CACGTG, however differ in the matrix similarity (MSS) and core similarity scores (CSS). The PWMs were applied to genomic sequences of regulated genes (see Material and Method section for details and Supplementary Figure S1 and Supplementary Table S1). As shown in Table 1 the number of c-Myc binding sites differed amongst individual genes, nonetheless an average of 2.6 c-Myc binding sites was computed (Supplementary Table S2).

In an effort to construct regulatory gene networks, the GeneWays information was considered. The software provides a system for extracting, analyzing, visualizing and integrating molecular pathway data. A maximum radius of 4 steps upstream of the input data was fixed and a total of 7 master regulatory molecules and their associated networks could be defined. These were (1) the RNA binding protein heterogeneous nuclear ribonucleoprotein A1 (*Hnrnpa1*), (2) the pre-rRNA transcription and ribosome assembly factor nucleolin (*Ncl*), (3) the multifunctional DNA repair enzyme *Apex1*, (4) the enzyme triosephosphate isomerase 1 (*Tpi1*) which catalyzes the isomerization of glyceraldehyde 3-phosphate and dihydroxyacetone phosphate in glycolysis and gluconeogenesis, (5) *Gapdh*, that is critically involved in the glycolytic pathway, (6) the folate transporter *Slc19a1* and (7) nucleophosmin (*Npm1*), i.e. a protein with diverse functions in ribosome biogenesis, histone assembly, cell proliferation and regulation of the p53 tumor suppressor. In Supplementary Figures S2–S8 individual networks are depicted and out of 87 up-regulated genes a total of 30, 34, 19, 31, 37, 37 and 35 were connected in the regulatory networks of *Hnrpa1*, *Apex1*, *Tpi1*, *Slc19a1*, *Ncl*, *Npm1* and *Gapdh*, respectively. Note the entire networks consisted of 60, 70, 37, 60, 78, 75 and 76 genes, respectively and the master regulatory molecules are supported by c-Myc DNA binding activity data obtained at gene specific promoters in EMSA assays while the quantitative metrics of the networks are given in Supplementary Table S3.

**Figure 2:**
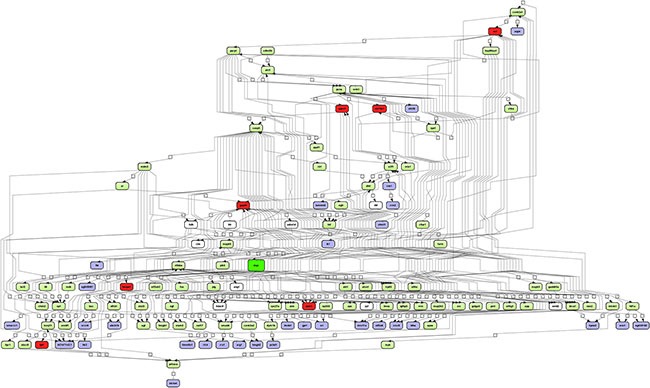
Integrated master regulatory gene network in PLACs of c-Myc transgenic mice The master regulatory networks of *Apex1, Gapdh, Hnrpa1, Ncl, Npm1, Slc19a1 and Tpi1* were fused. A total of 121 genes with connectivity to c-Myc signaling are depicted. Forty four percent of up-regulated genes are part of the network. Note, c-*Myc* itself is in the center of the network (color-coded in green) whereas regulated genes are given in blue and the master regulators are highlighted in red. Furthermore, expressed but unchanged genes in PLACs are shown in green. Connecting genes not found in the present study are colored in white. The networks were constructed with the GeneXplain platform; activation, inactivation and regulation are denoted by the symbols 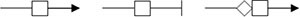, respectively.

In order to search for common crosstalk, the individual networks were fused and for 112 of the 121 network partners, transcript expression in PLACs was observed (Figure 2). The network comprised 38 up-regulated genes and included the master regulatory molecules (marked in red) *Hnrpa1*, *Apex1*, *Tpi1*, *Slc19a1*, *Ncl*, *Npm1* and *Gapdh* whose expression was induced by 2.6, 5.5, 2.8, 9.4, 3.2, 3.6 and 3.1-fold, respectively when compared to non-transgenic controls.

### Protein interaction networks

**Figure 3:**
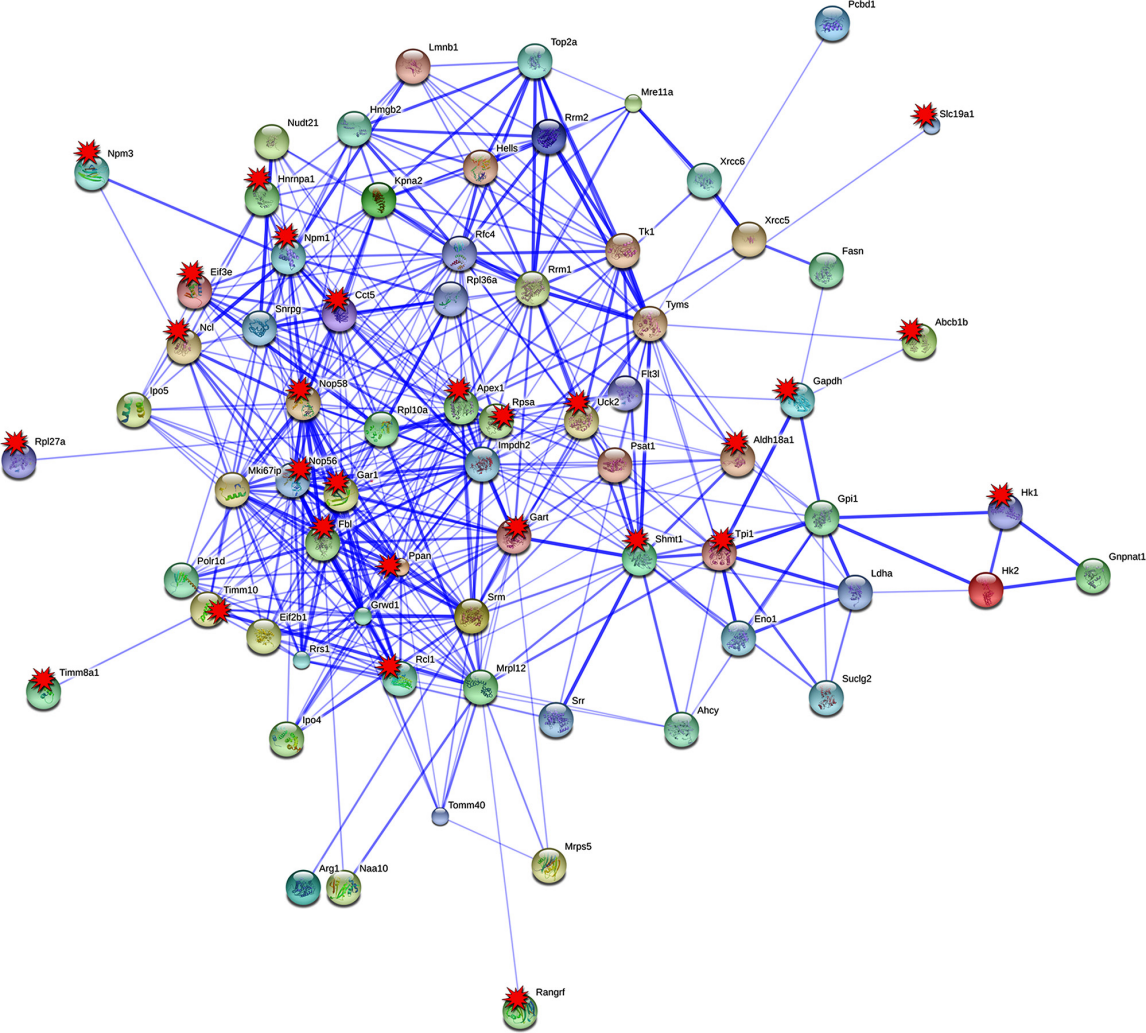
Protein interaction networks of in PLACs of c-Myc transgenic mice Protein-protein interaction (PPI) network were constructed with the STRING version 9.05. Eighty percent of up-regulated tumor genes were found to have 324 PPI interactions. EMSA confirmed c-*Myc* target genes are tagged with red colored multi-pointed star.

**Figure 5:**
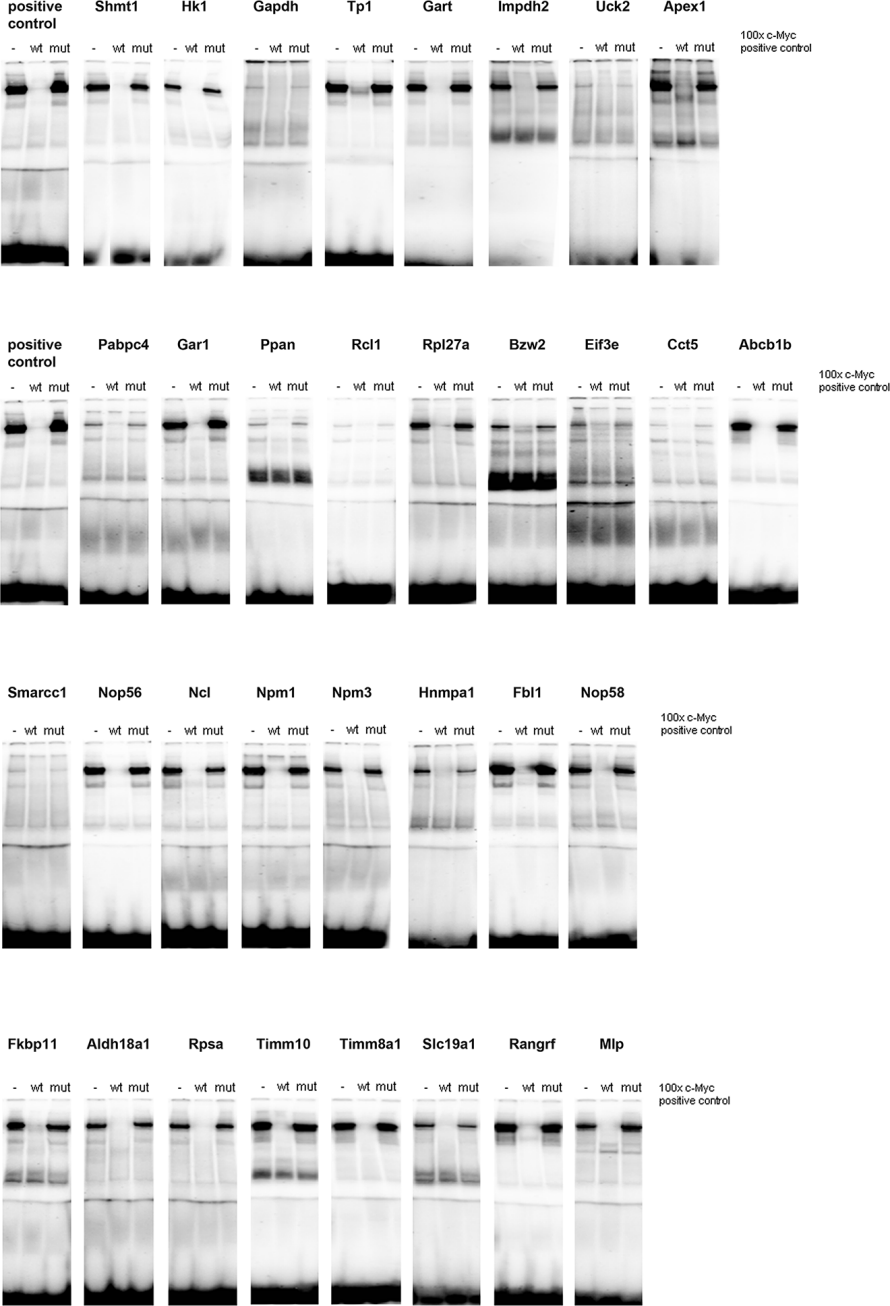
c-Myc DNA binding activity at gene specific promoters A total of 33 c-Myc binding sites were studied by EMSA band shift assay as described in Material and Method section and in Supplementary Table S2. Nuclear extracts of HeLa cells were used as a positive control. Depicted are EMSA assays with reference probes in competition assays, i.e. 100-fold excess. Specificity was assessed in competition assays with competitor probes where 1 nucleotide of the core consensus sequence is mutated (Supplementary Table S6 for probe sequences).

Based on STRING analysis and the information given in Table 1, a total of 70 PLAC regulated genes, and their coded proteins actually interacted with each other. Overall, 324 protein interactions were predicted, and for 27 PPI (Protein-Protein-Interaction) network partners, c-Myc DNA binding activity was corroborated at their gene specific promoters by EMSA (marked by a star in Figure 3 and presented in Figure 5), therefore reinforcing the strength of association among these network partners.

### Pathways mapping over protein network

**Figure 4:**
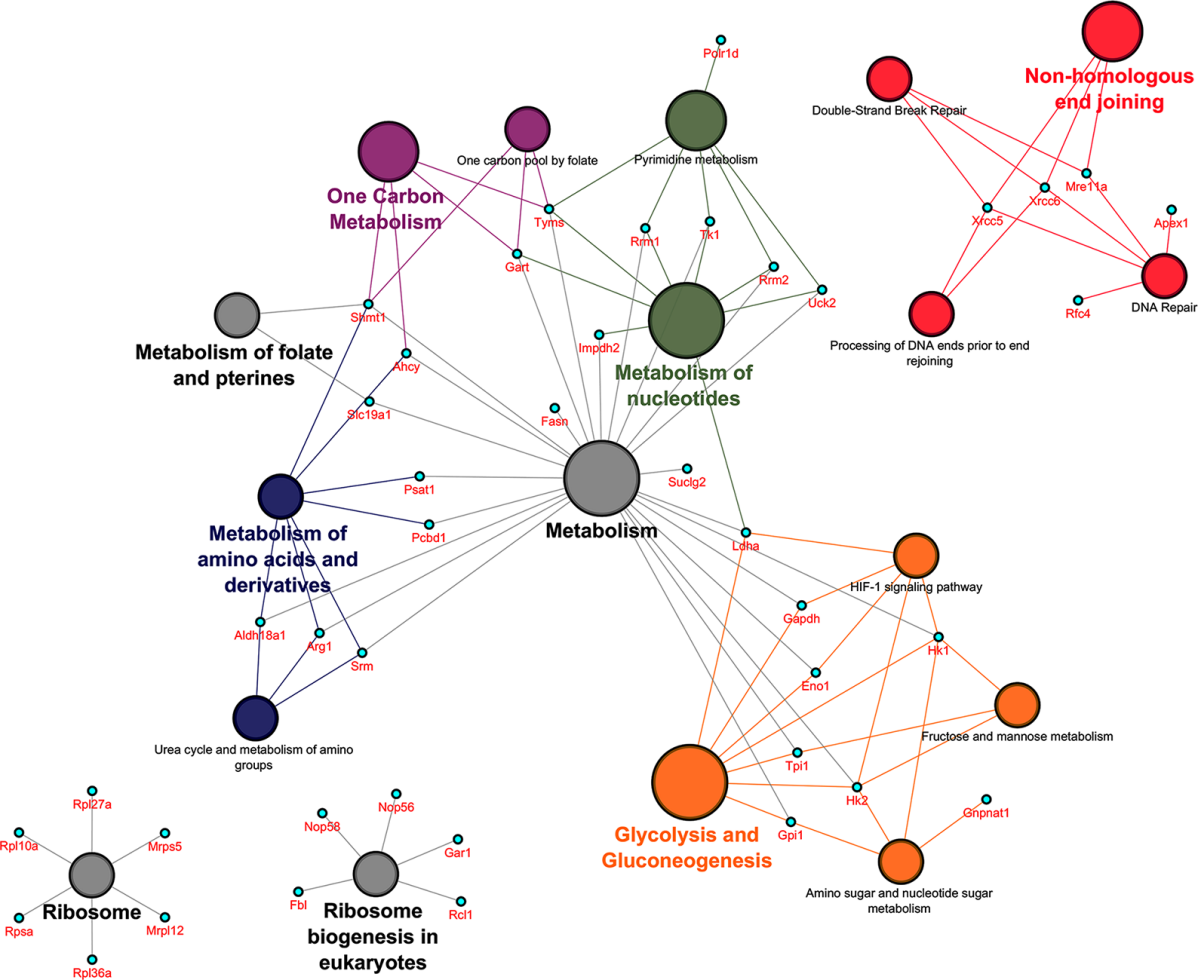
Pathway mapping over protein network in PLACs of c-Myc transgenic mice The Cytoscape version 3.0.2 with plugins (ClueGO v2.1.1 and CluePedia v1.1.1) was used to generate functional networks of biological pathways. Statistically significant pathway terms involved 42 out of 87 up-regulated genes. The symbol disc refers to a grouping of pathway terms and changes in its size imply an increase in statistical significance.

Out of the 70 PPI network partners 39 could be mapped to 5 distinct metabolic pathways, most notable glycolysis and gluconeogenesis (Eno1, Gapdh, Gpi1, Hk1, Hk2, Ldha, Tpi1), metabolism of nucleotides (Gart, Impdh2, Ldha, Rrm1, Rrm2, Tk1, Tyms, Uck2), metabolism of amino acids and derivatives (Ahcy, Aldh18a1, Arg1, Pcbd1, Psat1, Shmt1, Srm), one carbon metabolism (Ahcy, Gart, Shmt1, Tyms) and metabolism of folate and pterines (Shmt1, Slc19a1). Additionally, the pathway terms ribosome (Mrpl12, Mrps5, Rpl10a, Rpl27a, Rpl36a, Rpsa), ribosome biogenesis in eukaryotes (Fbl, Gar1, Nop56, Nop58, Rcl1) and DNA repair (Mre11a, Xrcc5, Xrcc6, Rfc4, Apex1) were significantly enriched (see Figure 4).

### Functional composite module construction

The co-occurrence of TF-binding sites at gene specific promoters was interrogated and the results are given in Supplementary Table S4. A composite module with a score of 27.68 and false positive and false negative call of 0 and 16%, respectively was computed and consisted of the position weight matrixes EBOX_Q6_01 and E2F_Q4_01 (see Supplementary Figure S9). Among 90 tumor regulated genes 95% had consensus binding sites for c-Myc (represented by PWM EBOX_Q6_01) while the combined EBOX_Q6_01 and TFDP1 (represented by PWM E2F_Q4_01) PWMs fitted 58 gene specific promoters or 65% of regulated genes (see Supplementary Table S5).

### c-Myc DNA binding activity at promoters of newly identified candidate genes

Given that the yield of nuclear protein from c-Myc transgenic lungs was extremely low, c-Myc DNA binding activity was investigated in gel shift assays using nuclear extract from c-Myc transgenic livers. In EMSA competition assays bands could be shifted with 100 times unlabeled probe, but not with a probe where a single base of the core consensus sequence was mutated (mut). For all of the 33 predicted genomic binding sites c-Myc binding activity could be demonstrated (Figure 5) and the oligonucleotide probes were designed on the basis of gene specific promoters sequences surrounding the E-box motive. c-Myc DNA binding activity differed amongst individual genes and the short naked ds-DNA sequences are given in Supplementary Table S6. Specifically less c-Myc DNA binding activity was observed at gene specific promoters of glyceraldehyde-3-phosphate dehydrogenase, uridine-cytidine kinase 2 (*Uck2*), poly(A) binding protein, cytoplasmic 4 (*Pabpc4*), RNA terminal phosphate cyclase-like 1 (*Rcl1*), chaperonin containing TCP1, subunit 5 (*Cct5*) and the SWI/SNF related, matrix associated, actin dependent regulator of chromatin, subfamily C, member 1 (*Smarcc1*).

### Gene reporter assays

Reporter assays were developed for the genes *Rcl1*, *Rpsa*, *Npm3* and *Hk1* which were predicted to contain 2, 2, 1 and 3 c-Myc binding sites, respectively, and had been confirmed in EMSA assays. Specifically, 2 Kb fragments of the mouse gene promoters were cloned into a dual luciferase retroviral vector [12] and transfected into human (HEK293T and SK-BR3) as well as mouse lung cancer cell line derived from a c-Myc/c-Raf double transgenic lung cancer model previously developed by us [17]. Thereafter, cells that express only low levels of endogenous c-Myc (HEK293T) were transfected with either an empty expression vector or with a c-Myc expression plasmid. Twenty-four hours after treatment with the c-Myc plasmid the cells were lysed and assayed for dual luciferase activity.

**Figure 6:**
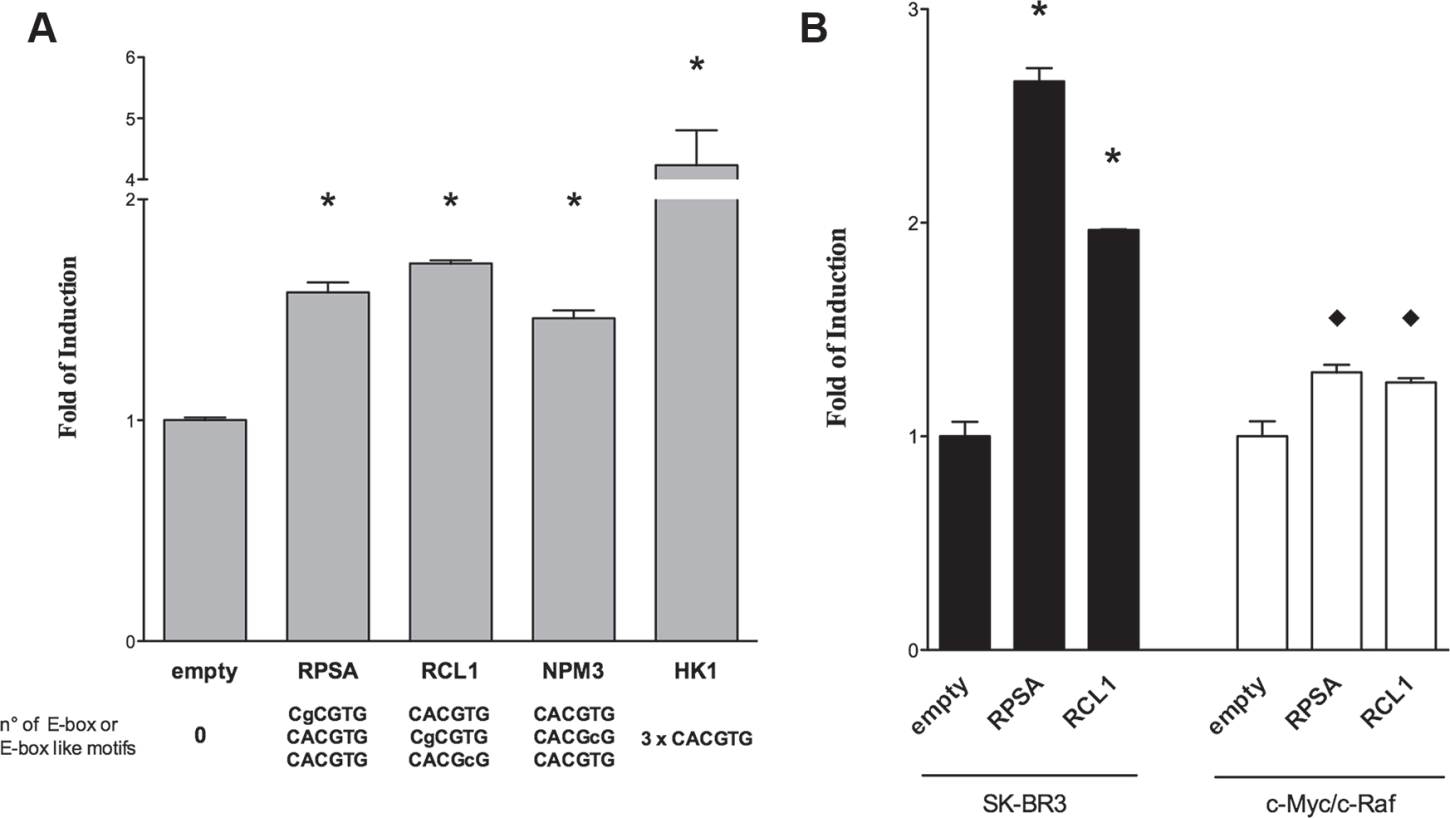
Gene reporter assays in HEK293T cells Dual-luciferase assays were performed in (**A**) HEK 293T and (**B**) human SK-BR3 or c-Myc/c-Raf mouse transgenic lung cancer cells transiently transfected with the pCZ-REN-P-LUC retroviral vectors containing the constitutive Renilla reporter and the Firefly reporter under the control of the gene specific promoter of the respective candidate genes. A c-Myc over-expression plasmid (MIG-MYC) was used in co-transfection assays to modulate c-Myc expression and to evaluate the impact on the gene reporter induction. Presented are the average ratios of the fold of reporter induction obtained in cells with ectopic over-expression of *c-Myc* compared to control cells with endogenous *c-Myc* expression. Error bars represent the standard deviation of at least three biological repeats. Student's *t*-test significance: * = *p* < 0.01; ♦ = *p* < 0.05.

In HEK293T cells results showed an increase in luciferase activity for all reporter constructs containing the c-Myc-dependent promoters (Figure 6A). As specified below the bars all promoter fragments harbored at least three different E-box-like sequences. Remarkably, the insertion of 2 Kb of the *HK1* promoter resulted in high induction of luciferase expression, possibly due to the presence of 3 consecutive repeats of the canonical E-box motives (CACGTG) within this promoter.

Subsequently the results were confirmed using two additional cellular systems (both human and murine) where c-Myc is expressed at high levels (Figure 6B). In HER2 positive breast cancer cells, SK-BR3 (black bars), *Rpsa* and *Rcl1* reporter constructs exhibited a strong increase in transactivation of the reporter gene as compared to the empty vector. This was further confirmed in a lung cancer derived murine c-Myc/c-Raf transgenic cell line with high expression level of c-Myc (white bars) where a modest but significant increase in reporter activity was observed. Given that the c-*Myc* transgene resulted in high constitutive expression levels our findings for *Rpsa* and *Rcl1* were not unexpected.

Taken collectively, an increased reporter activity for all the tested promoters was observed even in cells with abundant c-Myc expression. However, the level of induction differed among constructs and/or cell lines used (see Figure 6). This suggests that the number and/or position of the E-box site play a critical role in promoter activation.

### ChIP assays with HEK293T cells and ectopic c-Myc expression

**Figure 7:**
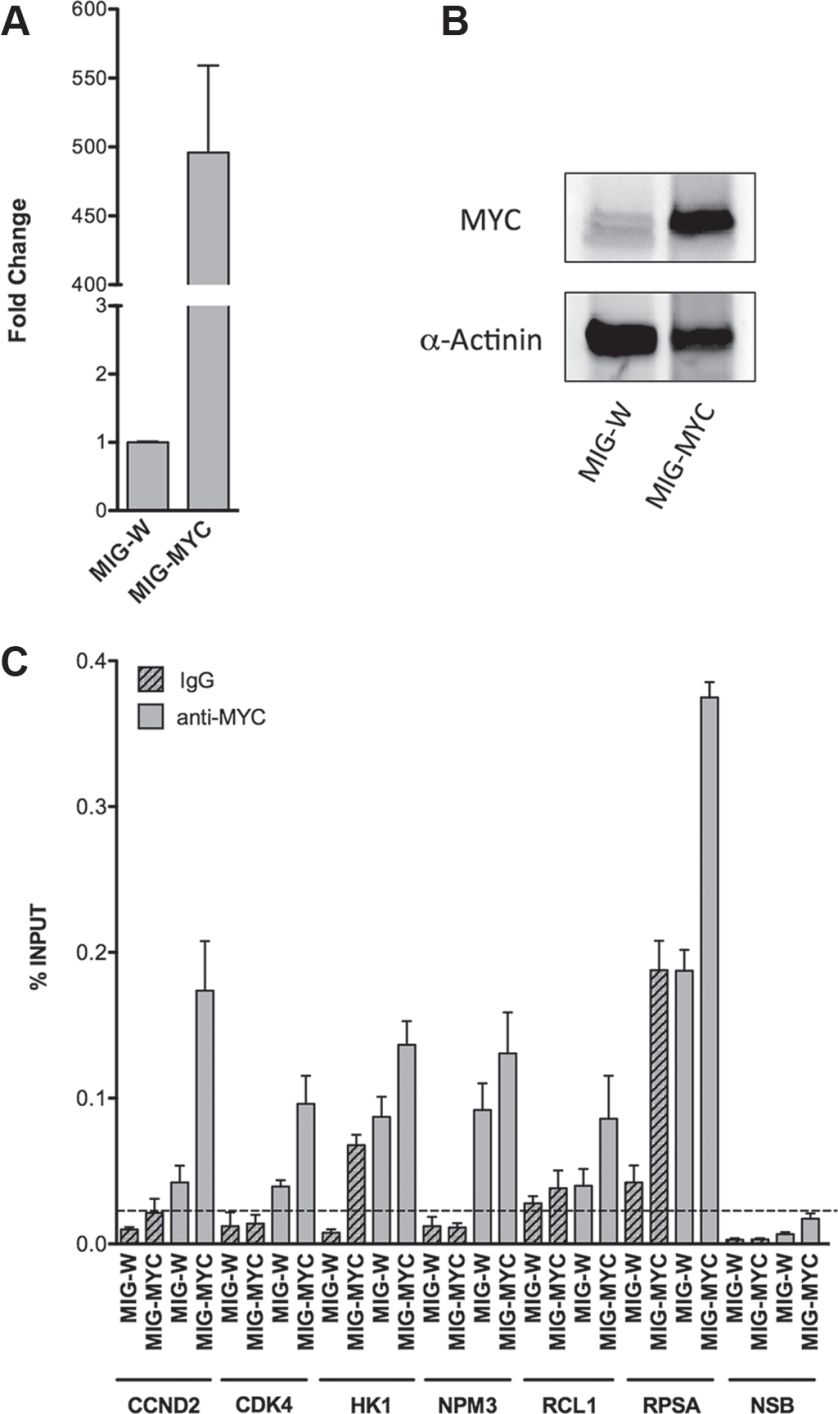
c-Myc over-expression and occupancy in HEK293T cells RT-qPCR (**A**) and Western blot (**B**) were performed to verify c-Myc increased levels upon over-expression in HEK293T cells. Cells were harvested 24 hours after c-Myc transient transfection (MIG-W represents the empty control). GAPDH and YWHAZ mRNAs and α-Actinin protein detection were used, respectively, as reference for qPCR normalization and loading control for Western blot. The bars in panel (A) indicate the average of two independent biological replicates with three technical repeats. The WB experiments shown in panel (B) are representative for one of two independent biological replicates. (**C**) ChIP assays were performed in HEK293T cells over-expressing c-Myc. qPCR quantification was performed with immuno-precipitated DNA of the anti-MYC monoclonal antibody (grey bars) or mouse normal IgG (grey patterned bars). To assay for Non Specific Binding (NSB) promoter regions of ACTB and the exon 9 locus of CCNB1 were amplified. Alike, qPCR of distinct CCND2 and CDK4 promoter regions served as positive controls. The level of non-specific occupancy by c-Myc over-expression is indicated with a dashed line. Plotted are the average levels of occupancy expressed as percentage of total input signals. Error bars represent the standard deviations of three technical replicates.

c-Myc occupancy at gene specific promoters of PLAC regulated genes was confirmed in ChIP assays. For this purpose HEK293T cells were transfected with a c-Myc containing plasmid. Ectopic c-*Myc* expression was confirmed by qRT-PCR and Western blot experiments. A remarkable increase in c-*Myc* transcript (Figure 7A) and protein (Figure 7B) expression was observed after transient transfection of the plasmid for 24 hours. c-Myc enriched DNA fragments were studied by qPCR using primers to amplify gene specific promoter regions of the c-Myc targets *RCL1*, *NPM3*, *HK1* and *RPSA*. The results were compared to mouse IgG which served as negative control. This revealed enrichment of c-Myc occupancy for the chosen and newly identified targets. The c-Myc recruitment on chromatin was comparable to positive controls (CCND2 and CDK4) where an increase is evident upon c-Myc over-expression (Figure 7C). Notably, the signal for *RPSA* was already high with IgG control that was similar to endogenous c-Myc binding in basal condition, nonetheless was further increased upon c-Myc over-expression.

### Chromatin-immunoprecipitation sequencing of human and murine cell lines

ChIP-seq data deposited within the ENCODE database of the UCSC Genome Browser (http://genome.ucsc.edu/ENCODE/) were analyzed by considering the human (hg19) and murine (mm19) datasets. Data from 7 human cell lines were analyzed for c-Myc binding and included the lymphoplastoid (GM12878), leukemia (K562), embryonic stem cell (H1-hESC), endothelial cell (HUVEC), hepatoma (HepG2), breast cancer (MCF-7) and cervical carcinoma (HeLa) cell lines. The same analysis was also performed using 2 murine cell lines (B-cell lymphoma -CH12-, analog of human GM12878 cell line and leukemia –MEL-, analog of human K562 cell line). The data were interrogated for PLAC regulated genes as identified in c-Myc transgenic mice (see Table 1) and the comparison was based on overlapping promoter sequences for independent experiments. As shown in Supplementary Table S7 ChIP-seq data from human as well as murine cell lines confirmed c-Myc binding for 94% and 91% of the up-regulated genes seen in transgenic PLAC. Fortuitously, the EMSA data obtained with nuclear extracts of the positive control (HeLa cells) could be directly compared with the ChIP-seq data for HeLa cells deposited in the ENCODE database. The data were in agreement, i.e. strong c-Myc binding sites seen in EMSA assays with mouse gene specific promoter sequences were likewise confirmed in ChIP-seq experiments using HeLa cells. Again the comparison is based on overlapping promoter sequences for independent experiments. Despite difference in the molecular organization of the mouse and human gene promoters there was considerable agreement in c-Myc binding amongst orthologous genes with ChIP-seq data of HeLa cells providing additional evidence for evolutionary conservation of c-Myc regulatory elements in 23 out of 33 EMSA assays performed (Figure 5). Given the considerable agreement between the gene expression, EMSA and ChIP-seq data derived from different cell lines and species, the results suggest high conservation of c-Myc DNA binding activity.

To further validate the EMSA findings (Figure 5) we interrogated the data reported by Sabo et al., 2014 [18] who investigated the role of c-Myc in a murine model of B-cell lymphoma and that of Walz et al., 2014 [19] who studied the consequences of inducible expression and depletion of c-Myc in human cells and murine tumor models. Importantly, all genes studied by EMSA were equally regulated in the aforementioned studies. The RPKM data from RNA-seq of normal (spleen-derived B cells as controls denoted as C in Supplementary Table S8), pre-tumor (with no infiltration of peripheral lymph nodes denoted as P) and tumor cells (lymphomas, denoted as T) was considered and calculated as averages from 4 samples each per condition to determine fold changes in pre-tumor (P/C) and tumor cells (T/C).

In the Supplementary Table S8 ChIP-seq data from Sabo et al., 2014 [18] are also given and enrichment was calculated as log2(ChIP - Input) with ChIP and Input data being the numbers of reads within a given peak divided by total library reads (in millions). In this way enrichment with respect to Input was calculated as fold change. “0” indicates that no peak was observed at that gene.

The agreement between the EMSA findings reported by us with c-Myc occupancy data in mouse cancer tissues or cells from two independent studies [18, 19] supports the relevance of our findings.

### Translational research

**Figure 8:**
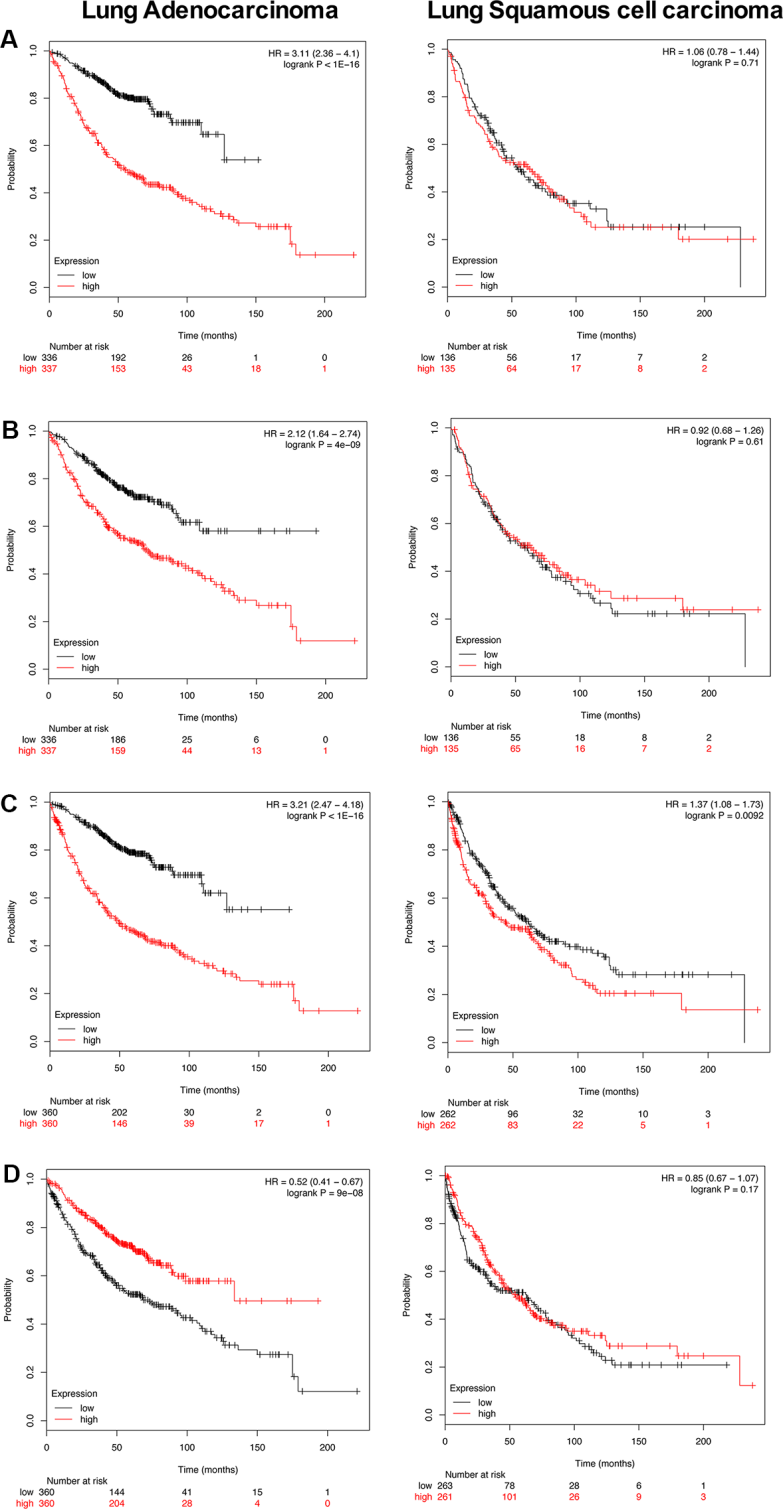
Kaplan-Meier survival plots Overall survival of PLAC regulated genes in human lung adenocarcinoma and squamous cell carcinoma patients was computed using the KM plotter online tool (http://kmplot.com/analysis/) [87]. The plots present survival curves according to their low and high expression. Fifty out of 90 regulated genes (47 up- and 3 down-regulated) were significantly associated with survival of lung cancer patients thus adding clinical relevance to our findings (Supplementary Table S9). (**A**) Depicted is the survival plot for 15 PLAC regulated genes. When studied individually each gene is associated with a HR ≥ 2. Their high expression defines poor outcome in lung adenocarcinoma (left panel; HR 3.11 *p* < 0.001) but not in squamous cell carcinoma patients (right panel; HR 1.06 *p* = 0.71) and the analysis is based on 673 and 270 patients, respectively. (**B**) Shown is the survival plot for 16 up-regulated PLAC genes with a FC > 5. Their high expression is associated with poor outcome (left panel; HR 2.12 *p* < 0.001) in lung adenocarcinoma but not squamous cell carcinoma (right panel; HR 0.92 *p* = 0.61) patients. (**C**) High expression of the master regulators *GAPDH*, *TPI*, *NPM1* and *SLC19A1* is associated with poor survival in lung cancers (both for adenocarcinoma and squamous cell carcinoma patients). (**D**) High expression of *SABT1*, *ANP32A* and *HIST1H2BC* is associated with better survival (left panel; HR 0.52 *p* < 0.001) in human lung adenocarcinoma patients.

To explore clinical relevance of PLAC regulated genes survival statistics was computed using the KM plotter (http://kmplot.com/analysis/) [20]. Importantly, the background database encompasses microarray expression profiling of human lung tumors from 2,437 lung cancer patients. As the microarray platform is identical to the one used in the present study direct comparisons could be made. Altogether 87 up- and 3 down-regulated genes were considered of which 50 (47 up- and 3 down-regulated) were significantly associated with survival of lung cancer patients. Specifically, Figure 8A depicts the KM plot for genes found to be up-regulated in PLACs of c-Myc transgenic mice (Supplementary Table S9 informs on HR for individual genes in a cohort of 720 lung adenocarcinoma patients). Shown is the summary of a 15- gene signature and each of the genes fulfilled the criteria of HR ≥ 2. As shown in panel A high expression of these genes was associated with poor outcome in lung adenocarcinoma (HR 3.11 *p* < 0.001) but not so in squamous cell carcinoma patients (HR 1.06 *p* = 0.71). This analysis is based on 673 and 270 patients, respectively, and demonstrates specificity for lung adenocarcinomas. Similarly, we consider genes highly up-regulated in c-Myc transgenic tumors, i.e. ≥ 5 fold. This defined a 16-gene signature and once again their high expression was associated with poor outcome (left panel; HR 2.12 *p* < 0.001) in lung adenocarcinoma but not squamous cell carcinoma (right panel; HR 0.92 *p* = 0.61) patients (Figure 8B). We next computed survival statistics for the identified master regulators (presented in Supplementary Figures S2–S8 as individual networks) and found 4 out of 7 to be prognostic, i.e. *TPI1*, *GAPDH*, *SLC19A1* and nucleophosmin (*NPM1*). Once again, high expression of these master regulators was associated with poor survival in lung adenocarcinoma but not squamous cell carcinoma patients (Figure 8C; HR 3.2 *p* < 0.001). Lastly, in PLACs of c-Myc transgenic mice the genes *Satb1*, *Anp32a* and *Hist1h2bc* were repressed in expression. As shown in Figure 8D their high expression in lung adenocarcinoma patients was associated with better survival (HR 0.52 *p* < 0.001). Altogether the data shows relevance of the transgenic mouse model in recapitulating human lung adenocarcinoma.

## DISCUSSION

In an effort to define c-Myc oncogenic activity in papillary lung adenocarcinomas a transgenic disease model was investigated. This revealed regulatory gene networks linked to glycolysis and gluconeogenesis, nucleotide and ribosome biogenesis. The observed metabolic perturbations provide opportunities for the development of molecularly targeted therapies.

Specifically, the role of c-Myc in cancer cell energy metabolism was highlighted in the seminal review of Dang et al., 2009 [21]. There is conclusive evidence for c-Myc to stimulate transcription of glycolytic genes such as glucose transporter 1, hexokinase 2, fructose-6-phosphat-kinase and enolase 1. Changes in carbohydrate metabolism are commonly observed in tumors and are testimony to a highly conserved principle, whereby energy production relies on high rates of glycolysis even at physiological oxygen levels as compared to mitochondrial oxidative phosphorylation that is common to normal cells.

### Central role of c-Myc in cell metabolism

**Figure 9:**
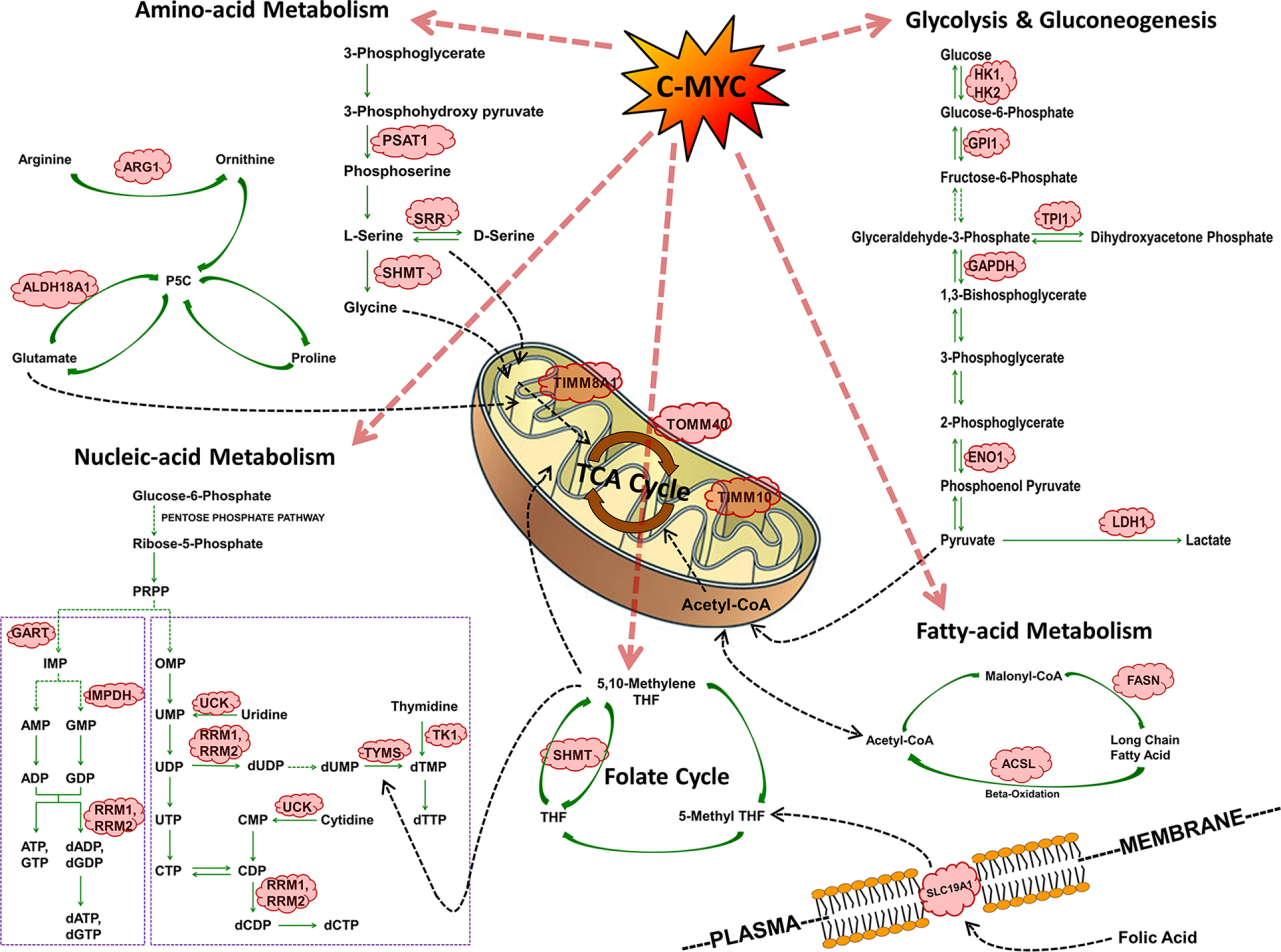
Summary of c-Myc regulated cell metabolism genes in PLAC

In lung tumors of c-Myc-transgenic mice induction of genes coding for carbohydrate, protein and nucleic acid metabolism was observed (see Table 1 and Figure 9). Nearly 54% of the regulated genes are known to be c-Myc-responsive when assessed amongst different cell lines and cell types. The fact that c-Myc binding sites are present in > 95% of up-regulated tumor genes (Table 1) is suggestive for c-Myc to directly activate their transcription. A comparison of overlapping promoter sequences between mouse tumor regulated genes and ChIP sequencing data for 7 different human and 2 murine cell lines confirmed 94% and 91%, respectively to be c-Myc bona fide targets (Supplementary Table S7), thus supporting the notion for c-Myc to be a key regulator of cell metabolism.

### Arginine auxotrophy

An important finding of the present study was the strong > 14-fold induction of arginase-1 in large papillary lung adenocarcinomas to suggest arginine dependent tumor growth. Arginase-1 is primarily expressed in liver and an essential component of the urea cycle, however is one of the 4 genes in Table 1 where computational analysis did not identify c-Myc binding sites even though tumor size dependent induction of the gene was observed. Moreover, high *Arg1* gene expression is associated with poor survival in human lung cancer patients (see Supplementary Table S9 and Figure 8). Importantly, targeting arginine-dependent cancers is the subject of intense research and arginine deprivation through induction of arginine deiminase will modulate the arginine cancer metabolome and therefore represents a novel antimetabolite strategy for the treatment arginine auxotrophy in lung cancers [22].

### Carbohydrate metabolism

Unlike normal cells and as a result of a metabolic switch that is referred to as the Warburg effect, tumor cells rely on glycolysis for energy production, even in a non-hypoxic environment [23]. Although less efficient, aerobic glycolysis is a rapid means of ATP synthesis to sustain tumor growth. As shown in Table 1 several genes regulated in PLACs code for glycolytic enzymes and contain c-Myc binding sites in their promoters. It is of considerable importance that glycolytic enzymes influence cell proliferation and have been shown to play decisive roles in tumor progression that is of diagnostic and therapeutic utility [24]. In the present study, mitochondrial hexokinase 2 was up-regulated by nearly 5-fold (Table 1) and was reported to inhibit apoptosis by preventing oligomerization of the pro-apoptotic BCL-2 family members BAX and BAK at the outer mitochondrial membrane [23, 25]. Furthermore, a recent study demonstrated HK2 to be essential for lung tumor initiation and maintenance; its systemic ablation conferred therapeutic efficacy in mouse models of lung cancer with *Hk2* deletion, suppressing glucose-derived ribonucleotide synthesis in lung cancer cells [26].

Likewise, glyceraldehyde-3-phosphate dehydrogenase and hexokinase 1 genes were up-regulated by > 3-fold in PLACs (Table 1) and were also reported to be up-regulated in human lung cancers [27, 28]. Apart from influencing glycolysis, GAPDH may possible participate in the regulation of telomeres [27]. Note, a recent study evidenced *Gapdh* over-expression to be a prognostic factor for poor outcome in NSCLC patients and was shown to correlate with fluorodeoxyglucose uptake in diagnostic PET imaging [29].

The regulation of glucose phosphate isomerase 1/autocrine motility factor in PLACs of transgenic mice further documents an eminent role of c-Myc in energy metabolism. Specifically, this multifunctional protein is a key player in glycolysis and acts in the extracellular milieu as a potent mitogen/cytokine. Its over-expression contributes to motility, invasion, and metastasis [23, 30] and was shown to correlate with an aggressive tumor growth and poor prognosis in human lung adenocarcinomas [23, 31]. Therefore, it has been explored as serum tumor marker in lung cancer patients [24].

Besides, hypoxia-inducible transcription factor signaling plays a critical role in glycolysis. It was established that c-Myc and HIF1 cooperate whereby HIF1 stimulates expression of glycolytic genes under hypoxic conditions whereas c-Myc regulates the same genes under non-hypoxic conditions thus contributing to the Warburg effect. Apart from responding to energy demands, this will influence nucleotide-, lipid- and amino acid synthesis that is of great utility for tumor growth.

For instance, fatty acid synthase (FASN), a key metabolic enzyme catalyzing the synthesis of long-chain saturated fatty acids, plays a central role in the production of surfactant in the lung. Over-expression of FASN has been shown to result in changes of membrane composition and to modulate lipid rafts in tumor cells. Lipid rafts are membrane microdomains involved in signal transduction, intracellular trafficking and cell migration [32], and FASN was reported to be induced at a high level in various human malignancies [33]. The ability of flavonoids to induce apoptosis in cancer cells is strongly associated with their capability to inhibit FASN [34].

In the present study the heat shock inducible transcription factor *Hif1a* was marginally induced (1.5-fold, *p* < 0.001, data not shown in Table 1 as it did not meet the threshold criteria), however, several *Hif1* responsive genes such as Hexokinase 2, triose phosphate isomerase 1, *Gapdh*, enolase 1, *Ldh1* and ribosomal protein SA were significantly induced by nearly 5-, 3-, 4-, 4-, 4- and 3-fold, respectively.

As shown in Table 1, lactate dehydrogenase 1 was up-regulated in PLACs. This enzyme catalyzes the conversion of L-lactate and NAD into pyruvate and NADH; its increased tissue expression was associated with poor outcome in lung cancer patients and was strongly associated with tumor recurrence, however did not correlate with NFκB p65 tissue expression or FDG uptake in PET imaging of NSCLC patients [35, 36]. HIF1A was also reported to influence apoptosis via a mechanism that involves the glycolytic pathway [37, 38].

A clinical trial amongst Chinese patients revealed enolase 1 protein levels in tumor tissues and circulating plasma samples of NSCLC patients to be increased to suggest this glycolytic enzyme to be of diagnostic utility [39]. In the present study enolase 1 expression was also up-regulated and found to be steadily increased from small to large PLACs. However, an earlier study reported enolase 1 to be down-regulated in NSCLC patients and the authors proposed a regulatory loop that involved the TATA-box binding protein (TBP) to confer c-Myc repression [40].

### Transporters, solute carriers and cancer metabolism

Altered metabolism in tumor growth and progressive disease has been the subject of independent reviews and the re-programming of metabolic pathways has important implications for diagnosis and the development of molecularly targeted therapies [24]. Several genes up-regulated in lung tumors of c-Myc transgenic mice code for cellular metabolism to support cell proliferation and included the multidrug resistance transporter *Abcb1b*, the oligopeptide transporter *Slc15a2*, the sodium bicarbonate co-transporter *Slc4a4* & *Slc4a7* and the folate transporter *Slc19a1*. A recent study reported the folate receptor alpha and *Slc19a1* to be over-expressed in NSCLC with implications for antifolate chemotherapy [41], while an earlier investigation suggested genetic variants in folate metabolism genes to be associated with risk of lung cancer among Chinese patients [42]. Outstandingly, serine hydroxymethyl transferase 1 (*Shmt1*) was nearly 10-fold up-regulated in PLACs and Western blotting confirmed induced protein levels (see Figure 1 panel C and D). This key enzyme of folate metabolism supplies one-carbon units for thymidylate biosynthesis and functions as a metabolic switch to priorities dTMP over SAM synthesis, i.e. a cofactor that methylate DNA, RNA, proteins, and many metabolites [43].

A case-control study with 1,032 lung cancer patients and 1,145 matched cancer-free controls suggested variants of serine hydroxymethyl transferase 1 to play a role in the etiology of lung cancer [44] and a recent study linked SNPs in folate metabolism genes to risk for lung cancer in never-smokers [45, 46]. While folate metabolism may affect the methylation status of tumor suppressor genes, the prognostic significance of functional SNPs in folate metabolism genes are insufficiently robust to distinguish individual patient outcome [47]. Moreover, serine hydroxymethyl transferase 1 knockdown induced apoptosis in lung cancer cells that was independent on serine or glycine starvation, but was the result of uracil disincorporation during DNA replication [48].

Additional genes up-regulated in folate mediated one-carbon metabolism were phosphoribosylglycinamide formyltransferase (*Gart*) and thymidylate synthase (*Tyms*), and are part of the de novo purine and pyrimidine biosynthetic pathways, respectively.

Remarkably, 9 genes coding for enzymes in purine and pyrimidine nucleotide biosynthetic pathways were up-regulated in PLACs (Table 1, *Tk1*, *Tyms*, *Shmt1*, *Srm*, *Impdh2*, *Gart*, *Uck2*, *Rrm2*, and *Rrm1*) with thymidylate synthase taking one a pivotal role in the maintenance of dTMP pool to support DNA replication. This enzyme catalyzes the transformation of deoxyuridylate to deoxythymidylate using 5,10-methylenetetrahydrofolate as a C1 donor. It also functions as an RNA binding protein and forms a complex with a number of cellular mRNAs including p53 to endorse its translational repression [49], thus influencing cell cycle and apoptosis. Besides, polymorphisms of thymidylate synthase contribute to risk for lung cancer and its interactions with dietary factors in lung cancer development [50]. Inhibition of thymidylate synthase by 5-fluorouracil is a well-established therapeutic strategy.

Furthermore, thymidine kinase 1 (*Tk1*), a gene coding for a key enzyme in the salvage pathways of DNA synthesis was induced > 4-fold in PLACs (Table 1, small tumors) and was reported to be a prognostic marker for NSCLC [51]. Note, uridine and thymidine kinase activities are strongly up-regulated in pulmonary cancers [52].

The ribonucleotide reductase *Rrm1* and *Rrm2*, which catalyzes the conversion of ribonucleoside 5′ -diphosphate into 2′ - deoxyribonucleoside 5′ -triphosphate were nearly 4- and 6-fold up-regulated at the transcript level in PLACs (Table 1) and are of critical importance in cell division and DNA repair [53]. Silencing of *Rrm1* and *Rrm2* markedly enhanced the cytotoxicity of the topoisomerase I inhibitor camptothecin that might be exploited in chemotherapeutic strategies [24, 54] and RRM2 was shown to regulate Bcl-2 in lung cancers and considered to be a worthy target in cancer therapy [55].

A further enzyme of the one-carbon metabolic pathway up-regulated was S-adenosyl homocystein hydrolase (*Ahcy*); its over-expression was similar among small, medium and large PLACs (Table 1). Importantly, the epigenetic anticancer drug 3-deazaneplanocin inhibits S-adenosyl homocystein and suppresses indirectly S-adenosyl methionine (SAM)-dependent cellular methylation reactions; based on its pharmacological mode of action it constitutes a new paradigm in the treatment of lung cancer [56].

Equally, the > 5-fold up-regulation of spermidine synthase (*Srm*) in large PLACs of c-Myc transgenic mice is a significant finding and there is growing evidence for c-Myc in regulating genes of polyamine metabolism as recently reported by us [12]. Specifically, polyamines take on multiple functions in cell proliferation and differentiation and their biosynthetic pathway involves decarboxylation of ornithine by ODC to yield putrescine while spermidine is formed by the addition of an aminopropyl group via the activity of spermidine synthase. In the present study the genes coding for ODC and SAM decarboxylase were significantly (*P* < 0.001) up-regulated by nearly 2-fold, however did not reach the threshold criteria (3-fold) set for Table 1. In a perspective article in clinical lung cancer the renaissance of polyamine metabolism inhibitors in cancer treatment was featured [57] and original research found *ODC* (SCL25A21) mRNA expression in lung tumors to be a prognostic factor in NSLC [58]. In addition, targeting *Srm* for the treatment of B-cell lymphomas is actively pursued [59] and polyamines have been shown to influence activity of NFκB that was associated with an up-regulation of genes involved in proliferation in breast cancer cells [60].

### Ribosome biogenesis

c-Myc is a regulator of ribosomal biogenesis [61] and several ribosomal proteins, e.g. *Rpsa*, *Rpl10a*, *Rpl27a*, *Rpl36a*, *Mrpl12* and *Mrps5*, were significantly up-regulated in lung tumors of c-Myc transgenic mice. The ribosomal protein SA, that functions as the lamin receptor in cell adhesion of the basement membrane, was reported to be up-regulated in NSCLC as evidenced by suppression subtractive hybridization of cDNA libraries generated from lung cancer patients [62]. Likewise, the ribosomal proteins S2 and L10a have been explored as tumor antigens for the development of peptide-based cancer immunotherapies [63], and phage display of tumor antigens yielded a panel of antigens specific to squamous cell carcinomas with silencing of ribosomal protein L23 inhibiting proliferation, invasion and cell survival in a mouse disease model [64]. In the same way the ribosomal protein L36A plays an important role in cell proliferation and may be exploited for the development of anticancer therapy [65].

The processing of pre-rRNA requires small nucleolar ribonucleoprotein particles (snoRNPs) in addition to trans-acting factors. Importantly, there are two classes of snoRNPs with the box C/D snoRNPs consisting of the core particles NHP2-like protein 1, NOP1/Fibrillarin, NOP56 and NOP58. Note, with the exception of the NHP2-like protein 1 where a minor but significant 1.5-fold increase (*p* < 0,001) was observed, the snoRNPs Fibrillarin/NOP1, NOP56 and NOP58 were up to 6-fold over-expressed in PLACs (Table 1). Additionally, the snoRNP box H/ACA *GAR1* and the RNA terminal phosphate cyclase-like 1 *RCL1* were up-regulated at the transcript level in lung tumors by 5- and 4-fold, respectively when compared to non-transgenic healthy controls. Thus, ribosome biogenesis is highly regulated in PLACs of c-Myc transgenic mice. A recent study on Burkitt's lymphoma implicated *Nol5a*/*Nop56* in oncogenesis [66]. As shown in Table 1, the genes coding for *Nol5a*/*Nop56* were highly significantly up-regulated in PLACs and *Nol5a* was shown to be necessary for c-Myc-induced cell transformation and tumor growth [66]. A recent study also suggests snoRNAs to have multiple functions in carcinogenesis with snoRNA42 acting as an oncogene in lung tumors [67], while whole-exome sequencing of DNA from two early onset lung adenocarcinoma never-smoking patients revealed germline mutations in *Nop58*, therefore adding weight to the role of snoRNPs in lung cancer [68].

Over-expression of the *SNRPG* transcript was also observed and the gene codes for one of the common proteins of small nuclear ribonucleoprotein particles to participate in RNA editing. The present study also evidenced a significant 4-fold up-regulation of nucleophosmin/B23 (*Npm1*) in PLACs. This nucleolar phosphoprotein functions as a molecular chaperone in ribosomal protein assembly and transport and prevents proteins to form aggregates within the nucleolus [69]. In the review of Grisendi and colleagues [69] loss of heterozygosity of chromosome 5q was reported for human NSCLC and it should be noted that gene locus of *Npm1* resides in this region [70] nonetheless, the protein is frequently over-expressed in solid tumors, while translocations are common to various leukemias [69]. Strong c-Myc DNA binding activity at promoter sites of *Npm1* and *Npm3* was observed in EMSA (Figure 5) as well as ChIP assays (Figure 7) and the results are confirmed in independent ChIP-seq experiments (Supplementary Table S7); equally our gene reporter assays (see Figure 6) confirm *Npm3* to be activated by c-Myc. Given its important role in cellular growth and proliferation we speculate *Npm1* to contribute to the onset of PLACs. Besides, this protein inhibits apoptosis by suppression of the protein kinase PKR in response to various extra- and intracellular signals [71].

Nucleolin is another nucleolar phosphoprotein over-expressed in PLACs and was shown to be a target of c-Myc [72]. Its translocation is regulated by heat shock cognate 70 to exert angiogenic functions [73] and controls c-Jun/Sp1-dependent transcriptional activation of cPLA2alpha in phorbol ester-treated non-small cell lung cancer A549 cells [74].

### DNA repair, genome stability and chromatin remodeling

Particularly with small PLACs, some highly over-expressed genes were identified and included topoisomerase (DNA) II alpha, ribonucleotide reductase M2, *Apex1*, *H1fx*, *Satb1* and *Rfc4* (Table1). Up-regulation of genes coding for the multifunctional DNA repair enzymes, such as *Apex1* and *Xrcc5*, is suggestive for c-Myc to influence DNA base excision repair and non-homologous end joining of DNA double-strand breaks. In support of angiogenesis and tumor progression, *Apex1* also enhances the DNA binding activity of a number of transcription factors and is considered to be a promising target for the consolidation of chemotherapy based on cisplatin [75].

The increased expression of linker histone *H1fx* and the repression of core histone *H2b1* suggest changes in the structure of nucleosome and altered access to nucleosomal DNA for gene expression, DNA replication and repair. Increased expression of helicase and *Smarcc1* was observed to effect DNA strand separation, gene transcription, DNA replication, recombination and repair. Conversely, *Satb1*, a key factor of chromatin remodeling and *Anp32a*, a tumor suppressor and inhibitor of histone acetyl-transferases, were repressed at the transcript level [76]. Note, it was recently demonstrated that microRNA-21 targets ANP32A [77] and their altered expression will effect chromatin remodeling to initiate undue expression of genes in response to c-Myc-hyperactivity.

### Regulation of orthologue genes in human malignancies

Several genes regulated in c-Myc transgenic PLACS contribute to cell growth, e.g. *Hk2*, *Fasn*, *Uck2*, *Impdh2* and *Tk1*, and were shown to be regulated in human malignancies as well [32, 78–80]. Importantly, altered expression of some of these orthologues was specifically reported for human NSCLC and included induction of *Rrm2* and *Top2a* with defined roles in DNA synthesis and transcription [81] as well as over-expression of *Arg1*, which fosters polyamine synthesis [82]. High activity of TYMS activity and GPI1 expression was associated with poor prognosis of patients with NSCLC [31, 83], as was increased activity of LDH with tumor stage [84]. Clearly, this demonstrates the relevance of the transgenic disease model for human NSCLCs. To the best of our knowledge, certain genes identified in the present study had not previously been associated with human lung carcinogenesis and translational research identified 47 PLAC up-regulated genes to be associated with poor outcome; conversely, high expression of the three repressed PLAC genes was associated with improved survival in lung adenocarcinoma but not squamous cell carcinoma patients (Figure 8, Supplementary Table S9). The Kaplan-Meier survival plots evidence clinical relevance of PLAC regulated genes in c-Myc transgenic mice with increased expression of master regulatory genes being associated with poor survival (HR 3.2, *p* < 0.001). Importantly, their inhibition opens new possibilities for therapeutic intervention strategies.

Taken collectively, oncogenomics combined with ChIP and EMSA as well as gene reporter assay and various computational methods revealed novel cell metabolism associated c-Myc regulated genes to broaden the perspective of molecularly targeted therapies for lung cancer.

## MATERIALS AND METHODS

### Ethics statement

All animal work strictly followed the Public Health Service (PHS) Policy on Humane Care and Use of Laboratory Animals. Formal approval to carry out animal studies was granted by the ethical review board of the city of Hannover, Germany. The approval ID is Az: 33.9-42502-04-06/1204.

### Maintenance of the transgenic mouse line

The development of the SPC/c-Myc-transgenic disease model was previously reported [11]. Mice were maintained as hemizygotes in the CD2F1-(DBA/2xBalb/C) background and the transgene was verified by PCR with DNA extracted from tail biopsies using Platinum PCR SuperMix (InVitrogen, Life Technologies, Darmstadt, Germany) and the following primer pair: 5′-CAGGGCCAAGGGCCCTTGGGGGCTCTCACAG, 3′-GGACAGGGGCGGGGTGGGAAGCAGCTCG.

### Sample collection and preparation

Mice aged between 9–13 months were anesthetized by an overdose of CO_2_ and the lungs were explanted and rinsed with ice cold physiological saline. The tumors were inspected macroscopically, separated from the surrounding lung tissue and frozen immediately in liquid nitrogen. The tumors were divided into groups according to size (1 mm, 5 mm, and > 10 mm). Small tumors were pooled in three groups of 3, 3, and 4 separate tumors because of the low yield of RNA. Normal lungs from *n* = 4 non-transgenic mice of about the same age were used as control.

### Isolation of RNA, array hybridization, scanning

Total RNA was isolated with the Qiagen RNA purification kit according to the manufacturer's instructions (Qiagen, Hilden, Germany); RNA integrity and quality was studied with the Agilent Bioanalyzer 2100 (Agilent Technologies, Santa Clara, CA, USA). Whole genome transcript profiling was performed with the Affymetrix Murine Genome U74v2 GeneChip expression oligonucleotide array as recently reported and the data were deposited in the public domain (accession number GSE54829) [12].

### Bioinformatics analyses

c-Myc binding sites, co-occupancy of additional transcription factors and the identification of master regulators was computed with the GeneXplain platform, Transfac(R), the MATCHTM algorithm and Gene Ways network as recently described [12]. See Supplementary Tables S3, S4 and S10, for additional details.

### Protein network construction and pathway mapping over protein network

Protein-protein interactions amongst DEGs were investigated using the STRING (http://string-db.org/) software version 9.05. Only proteins of *M. musculus* were considered and the confidence scores for each interaction pair was set > 0.4.

Pathways mapping over protein network was performed with the Cytoscape software version 3.0.2 using ClueGO and CluePedia as plugins. Additional information from KEGG, REACTOME and WikiPathways databases were retrieved and ontology terms with a statistical significance of *p* < 0.05 were used for further studies (see Supplementary Table S11).

### Gene expression studies by RT-PCR

Total RNA was isolated with the Qiagen RNA purification kit according to the manufacturer's instructions. Reverse transcription was carried out using Omniscript (Qiagen), Oligo-dT primers (InVitrogen, Life Technologies), and RNasin (Promega, Mannheim, Germany), followed by PCR amplification (see above) with the primer pairs given in Supplementary Table S12.

Semi-quantitative PCR reactions were done with Taq Platinum PCR Super-Mix Kit (In Vitrogen, Life Technologies), and amplification products were separated on 1% agarose gels. Densitometric scans were obtained the with Kodak 1D Image Analysis Software. The gene expression values were normalized to beta-actin expression. The fold change was computed as a ratio between gene expression values for tumor and control non-transgenic lungs.

### Electromobility band shift assays

The oligonucleotides were purchased from MWG Biotech (Ebersberg/Muenchen, Germany) and were used as double-stranded 32P-labeled probes as previously reported [17] For sequence information see Supplementary Table S6.

Oligonucleotides were annealed at a final concentration of 1 nmol in a buffer containing 20 mM Tris (pH 7.6), 10 mM MgCl_2_ and 50 mM NaCl at 80°C for 10 min and then cooled slowly to room temperature overnight and were stored at 4°C. Annealed oligonucleotides were diluted to 1:10 in Tris-EDTA buffer (1 mM EDTA, 10 mM Tris, pH 8.0) and 4 pmol were labeled using [32P] ATP (Perkin, Elmer, Rodgau-Jügesheim, Germany) and T4 polynucleotide kinase (New England Biolabs GmbH, Frankfurt am Main, Germany). End-labeled probes were separated from unincorporated [32P] ATP with a Microspin G-25 Column (GE Healthcare Europe GmbH, Freiburg, Germany) and eluted into a final volume of 100 μL.

5–10 μg nuclear extract and 10^5^ cpm labelled oligonucleotides were incubated in binding buffer consisting of 25 mM HEPES (pH 7.6), 5 mM MgCl_2_, 34 mM KCl, 2 mM DTT, 2 mM Pefablock (Roche Diagnostics GmbH, Mannheim, Germany), 2% aprotinin (Sigma-Aldrich Chemie GmbH, Taufkirchen, Germany), 40 ng poly (dl-dC)/μl and 100 ng bovine serum albumin/μl (PAA Laboratories GmbH, Cölbe, Germany). The binding of nuclear protein was allowed for 20 min on ice and free DNA and DNA-protein complexes were resolved on a 6% polyacrylamide gel. Competition studies were done by adding a 100-fold excess of unlabeled wild type or mutant oligonucleotides to the reaction mix. Gels were blotted to Whatman 3 MM paper, dried under vacuum, exposed to imaging screens (Imaging Screen-K, Bio Rad Laboratories GmbH, München, Germany) for autoradiography overnight at room temperature and analyzed using a phosphor imaging system (Molecular Imager FX pro plus; Bio Rad Laboratories GmbH, München, Germany) and the Quantity One Version 4.2.2 software (Bio Rad Laboratories GmbH, München, Germany).

### Gene reporter assays

HEK 293T and SK-BR3 cells were purchased from the American Type Culture Collection (ATCC) and cultured in DMEM medium containing 10% FBS (PAA, Coelbe, Germany), L-Glutamine and antibiotics. Starting from genomic DNA, a 2 Kb gene specific promoter region for mouse *Rcl1, Rpsa, Npm3* and *Hk1* genes (centered around the TSS) was cloned into the pCZ-REN_LUC retroviral plasmid using a PCR approach as recently described [12, 85].

Briefly, a two Kb fragments of the chosen promoters were PCR amplified from mouse genomic DNA with pair of primers containing restriction sites, sub-cloned into the pCR4-TOPO vector and thereafter cloned into pCZ-REN_LUC at the BamHI and Hind III sites (sequences of the primers used are available upon request). The genes *Rpsa, Npm3, Rcl1* and *Hk1* were studied in the HEK 293T or SK-BR3 cell lines. Correct cloning into pCZ-REN-P-LUC plasmids was confirmed by restriction analysis and DNA sequencing.

Respectively, 1.2–1.5 × 10^5^ HEK 293T cells and 1 × 10^5^ SK-BR3 cells were seeded onto 24-well plates and transfected at ∼80% confluence with Lipofectamine 2000 according to the manufacturer's instruction (InVitrogen, Life Technologies). The direct role of c-Myc activity was tested by co-transfecting the cells with or without a c-Myc over-expressing vector (MIG-MYC); the amount of transfected DNA amount was kept constant adding an equal volume of an empty vector (MIG-W). Twenty-four hours after transfection dual luciferase assays were done, following the manufacturer's protocol. A multilabel plate reader for the detection of light signals was used (Victor3, Perkin Elmer, Nürnberg, Germany).

### Chromatin immuno precipitation (ChIP) assays

ChIP assays were performed with the human HEK293T cells transiently over-expressing c-Myc. Cells were cultured in 150-mm dishes and transiently transfected with MIG-MYC or MIG-W empty vectors using Lipofectamine LTX according to manufacturer's instructions (InVitrogen, Life Technologies). After 24 hours, at near confluence (80–90%) formaldehyde (1%) was added to the cell culutre in order to cross-link proteins with DNA. Chromatin ImmunoPurification was carried out as previously described [86] using a Q900 sonicator with a plate horn (QSonica, Newtown, CT, USA), Protein G magnetic Dynabeads (Invitrogen, Life Technologies), 1 μg of anti-MYC monoclonal antibody (clone : 9E10, Santa Cruz Biotechnology, Milan, Italy) or 1 μg of mouse IgG as a negative control and eluted DNA was then purified with the QIAquick PCR purification kit (Qiagen, Milan, Italy). Immunoprecipitated chromatin was analyzed for c-Myc recruitment on selected genes (*RPSA*, *HK1*, *NPM3* and *RCL1*) by quantitative PCR (qPCR). Enrichment of c-Myc binding was calculated as percentage of Input DNA using the ΔCt method. qPCR was performed with the KAPA SYBR Green Universal qPCR mix (Kapa Biosystems, Resnova, Rome, Italy) employing standard conditions. Two different DNA loci were used as ChIP positive controls (*CCND2* and *CDK4* promoter regions). Alternatively, *ACTB* promoter and *CCNB1* exon 9 regions served as negative controls. Importantly primers for ChIP-PCRs were designed in such a way that regions of ChIP-seq peaks for c-Myc binding as reported in the ENCODE database were amplified (see below). Sequences of primers are presented in Supplementary Table S13.

### ChIP-seq data retrieval from the UCSC genome browser

c-Myc ChIP-seq data deposited in the UCSC Genome Browser (http://genome.ucsc.edu/) was retrieved from ENCODE (hg19 or mm9 for human or mouse genomic data, respectively) as previously described [12]. A total data of 7 human cell lines derived from 8 different experiments and 2 murine cell lines were analyzed. As shown in Supplementary Table S7, ChIP-seq data confirmed c-Myc binding for 93% (human cell lines) and 91% (murine cell lines) of the 87 up-regulated genes. Note, there were only 3 down-regulated genes rendering such analysis impossible.

### Western blotting experiments

Proteins from lung tumors of SPC/c-Myc-transgenic mice and/or non-transgenic animals were extracted by sonication in 500 μl benzonase containing 2D-loading buffer and stored at –80°C. The protein concentration was determined with the Bradford assay according to the manufacturer's recommendations.

Seventy-five or 100 μg of total protein extracts (50 μg in case of HEK293T transiently transfected with c-Myc vector) were separated on 12.0% SDS-polyacrylamide gel and blotted onto PVDF membranes in 25 mM Tris and 190 mM glycine at 4°C for 2 h at 350 mA or using the semi-dry iBlot transfer system (InVitrogen, Life Technologies). Specific antibodies were purchased from Santa Cruz Biotechnology, Inc. and diluted accordingly: Arg1 (1:100), Shtm (1:100), HxkII (1:100), Fasn (1:100), c-Myc (1:1000) and α-Actinin (1:8000). Antigen-antibody complexes were visualized using the ECL detection system NEN Life Science Products (PerkinElmer Life Science, Rodgau-Juegesheim, Germany) or ECL plus kit (GE-Healthcare, Milan, Italy) and a ChemiDoc XRS+ (BioRad, Milan, Italy) as recommended by the manufacturer and recorded with Kodak IS 440 CF (Kodak, Biostep GmbH, Jahnsdorf, Germany).

### Kaplan-Meier plots

Kaplan-Meier plots were generated as overall survival curves from lung adenocarcinoma and lung squamous cell carcinoma patients using the KM plotter online tool (http://kmplot.com/analysis/) [87]. The plots depict survival curves according to their low and high expression. Altogether 87 up- and 3 down-regulated genes were considered of which 50 (47 up- and 3 down-regulated) were significantly associated with survival of lung cancer patients. The KM plot informs on HR for individual genes in a cohort of 720 lung adenocarcinoma patients (Supplementary Table S9). Shown in Figure 8A is the summary of a 15-gene signature and each of the genes fulfilled the criteria of HR ≥ 2. The analysis is based on 673 adenocarcinoma and 270 squamous cell carcinoma patients, respectively. Similarly, ≥ 5 fold up-regulated PLAC genes were considered. This defined a 16-gene signature (Figure 8B). Survival statistics for master regulators (presented in Supplementary Figures S2–S8 as individual networks), i.e. TPI1, GAPDH, SLC19A1 and nucleophosmin was computed (Figure 8C). Lastly, KM plots for *SATB1*, *ANP32A* and *HIST1H2BE* were generated (Figure 8D). Research identified *SAT1B* to be a marker of poor survival and to support metastasis in small cell lung cancer [88, 89]. However, its high expression is associated with better survival in non-small lung cancer thus suggesting a specific role of this chromatin remodeling protein.

## SUPPLEMENTARY MATERIALS FIGURES AND TABLES

















## References

[1] Centers for Disease Control Prevention (2016). Fact sheet Smoking & Tobacco Use.

[2] World Health Organization

[3] Charloux A, Quoix E, Wolkove N, Small D, Pauli G, Kreisman H (1997). The increasing incidence of lung adenocarcinoma: reality or artefact? A review of the epidemiology of lung adenocarcinoma. International journal of epidemiology.

[4] Oster SK, Ho CS, Soucie EL, Penn LZ (2002). The myc oncogene: MarvelouslY Complex. Advances in Cancer Research.

[5] Geng Z, Zhang D, Liu Y (2003). Expression of telomerase hTERT in human non-small cell lung cancer and its correlation with c-Myc gene. Chinese medical journal.

[6] Tsai LH, Wu JY, Cheng YW, Chen CY, Sheu GT, Wu TC, Lee H (2015). The MZF1/c-Myc axis mediates lung adenocarcinoma progression caused by wild-type lkb1 loss. Oncogene.

[7] Romero OA, Torres-Diz M, Pros E, Savola S, Gomez A, Moran S, Saez C, Iwakawa R, Villanueva A, Montuenga LM, Kohno T, Yokota J, Sanchez-Cespedes M (2014). MAX inactivation in small cell lung cancer disrupts MYC-SWI/SNF programs and is synthetic lethal with BRG1. Cancer discovery.

[8] Sears RC (2004). The life cycle of C-Myc: from synthesis to degradation. Cell cycle (Georgetown, Tex).

[9] Fernandez PC, Frank SR, Wang L, Schroeder M, Liu S, Greene J, Cocito A, Amati B (2003). Genomic targets of the human c-Myc protein. Genes & development.

[10] Patel JH, Loboda AP, Showe MK, Showe LC, McMahon SB (2004). Analysis of genomic targets reveals complex functions of MYC. Nature reviews. Cancer.

[11] Ehrhardt A, Bartels T, Geick A, Klocke R, Paul D, Halter R (2001). Development of pulmonary bronchiolo-alveolar adenocarcinomas in transgenic mice overexpressing murine c-Myc and epidermal growth factor in alveolar type II pneumocytes. British journal of cancer.

[12] Ciribilli Y, Singh P, Spanel R, Inga A, Borlak J (2015). Decoding c-Myc networks of cell cycle and apoptosis regulated genes in a transgenic mouse model of papillary lung adenocarcinomas. Oncotarget.

[13] Menssen A, Hermeking H (2002). Characterization of the c-Myc-regulated transcriptome by SAGE: identification and analysis of c-Myc target genes. Proceedings of the National Academy of Sciences of the United States of America.

[14] Zeller KI, Jegga AG, Aronow BJ, O'Donnell KA, Dang CV (2003). An integrated database of genes responsive to the Myc oncogenic transcription factor: identification of direct genomic targets. Genome biology.

[15] Zeller KI, Zhao X, Lee CW, Chiu KP, Yao F, Yustein JT, Ooi HS, Orlov YL, Shahab A, Yong HC, Fu Y, Weng Z, Kuznetsov VA (2006). Global mapping of c-Myc binding sites and target gene networks in human B cells. Proceedings of the National Academy of Sciences of the United States of America.

[16] Reymann S, Borlak J (2008). Transcription profiling of lung adenocarcinomas of c-Myc-transgenic mice: identification of the c-Myc regulatory gene network. BMC systems biology.

[17] Reamon-Buettner SM, Borlak J (2008). Epigenetic silencing of cell adhesion molecule 1 in different cancer progenitor cells of transgenic c-Myc and c-Raf mouse lung tumors. Cancer research.

[18] Sabo A, Kress TR, Pelizzola M, de Pretis S, Gorski MM, Tesi A, Morelli MJ, Bora P, Doni M, Verrecchia A, Tonelli C, Faga G, Bianchi V (2014). Selective transcriptional regulation by Myc in cellular growth control and lymphomagenesis. Nature.

[19] Walz S, Lorenzin F, Morton J, Wiese KE, von Eyss B, Herold S, Rycak L, Dumay-Odelot H, Karim S, Bartkuhn M, Roels F, Wustefeld T, Fischer M (2014). Activation and repression by oncogenic MYC shape tumour-specific gene expression profiles. Nature.

[20] Szasz AM, Lanczky A, Nagy A, Forster S, Hark K, Green JE, Boussioutas A, Busuttil R, Szabo A, Gyorffy B (2016). Cross-validation of survival associated biomarkers in gastric cancer using transcriptomic data of 1,065 patients. Oncotarget.

[21] Dang CV, Le A, Gao P (2009). MYC-induced cancer cell energy metabolism and therapeutic opportunities. Clinical cancer research.

[22] Phillips MM, Sheaff MT, Szlosarek PW (2013). Targeting arginine-dependent cancers with arginine-degrading enzymes: opportunities and challenges. Cancer research and treatment.

[23] Garber K (2004). Energy boost: the Warburg effect returns in a new theory of cancer. Journal of the National Cancer Institute.

[24] Furuta E, Okuda H, Kobayashi A, Watabe K (2010). Metabolic genes in cancer: their roles in tumor progression and clinical implications. Biochimica et biophysica acta.

[25] Westphal D, Kluck RM, Dewson G (2014). Building blocks of the apoptotic pore: how Bax and Bak are activated and oligomerize during apoptosis. Cell death and differentiation.

[26] Patra KC, Wang Q, Bhaskar PT, Miller L, Wang Z, Wheaton W, Chandel N, Laakso M, Muller WJ, Allen EL, Jha AK, Smolen GA, Clasquin MF (2013). Hexokinase 2 is required for tumor initiation and maintenance and its systemic deletion is therapeutic in mouse models of cancer. Cancer cell.

[27] Guo C, Liu S, Sun MZ (2013). Novel insight into the role of GAPDH playing in tumor. Clinical & translational oncology.

[28] Smith TA (2000). Mammalian hexokinases and their abnormal expression in cancer. British journal of biomedical science.

[29] Puzone R, Savarino G, Salvi S, Dal Bello MG, Barletta G, Genova C, Rijavec E, Sini C, Esposito AI, Ratto GB, Truini M, Grossi F, Pfeffer U (2013). Glyceraldehyde-3-phosphate dehydrogenase gene over expression correlates with poor prognosis in non small cell lung cancer patients. Molecular cancer.

[30] Tsutsumi S, Yanagawa T, Shimura T, Kuwano H, Raz A (2004). Autocrine motility factor signaling enhances pancreatic cancer metastasis. Clinical cancer research.

[31] Takanami I, Takeuchi K, Naruke M, Kodaira S, Tanaka F, Watanabe H, Raz A (1998). Autocrine motility factor in pulmonary adenocarcinomas: results of an immunohistochemical study. Tumour biology.

[32] Swinnen JV, Van Veldhoven PP, Timmermans L, De Schrijver E, Brusselmans K, Vanderhoydonc F, Van de Sande T, Heemers H, Heyns W, Verhoeven G (2003). Fatty acid synthase drives the synthesis of phospholipids partitioning into detergent-resistant membrane microdomains. Biochemical and biophysical research communications.

[33] Evert M, Schneider-Stock R, Dombrowski F (2005). Overexpression of fatty acid synthase in chemically and hormonally induced hepatocarcinogenesis of the rat. Laboratory investigation.

[34] Brusselmans K, Vrolix R, Verhoeven G, Swinnen JV (2005). Induction of cancer cell apoptosis by flavonoids is associated with their ability to inhibit fatty acid synthase activity. The Journal of biological chemistry.

[35] Pullamsetti SS, Banat GA, Schmall A, Szibor M, Pomagruk D, Hanze J, Kolosionek E, Wilhelm J, Braun T, Grimminger F, Seeger W, Schermuly RT, Savai R (2013). Phosphodiesterase-4 promotes proliferation and angiogenesis of lung cancer by crosstalk with HIF. Oncogene.

[36] Nair VS, Gevaert O, Davidzon G, Plevritis SK, West R (2014). NF-kappaB protein expression associates with (18)F-FDG PET tumor uptake in non-small cell lung cancer:a radiogenomics validation study to understand tumor metabolism. Lung cancer (Amsterdam, Netherlands).

[37] Luo F, Liu X, Yan N, Li S, Cao G, Cheng Q, Xia Q, Wang H (2006). Hypoxia-inducible transcription factor-1alpha promotes hypoxia-induced A549 apoptosis via a mechanism that involves the glycolysis pathway. BMC cancer.

[38] Semenza GL (2003). Targeting HIF-1 for cancer therapy. Nat Rev Cancer.

[39] Zhang Y, Li M, Liu Y, Han N, Zhang K, Xiao T, Cheng S, Gao Y (2010). [ENO1 protein levels in the tumor tissues and circulating plasma samples of non-small cell lung cancer patients]. [Article in Chinese]. Zhongguo Fei Ai Za Zhi.

[40] Chang YS, Wu W, Walsh G, Hong WK, Mao L (2003). Enolase-alpha is frequently down-regulated in non-small cell lung cancer and predicts aggressive biological behavior. Clinical cancer research.

[41] Nunez MI, Behrens C, Woods DM, Lin H, Suraokar M, Kadara H, Hofstetter W, Kalhor N, Lee JJ, Franklin W, Stewart DJ, Wistuba II (2012). High expression of folate receptor alpha in lung cancer correlates with adenocarcinoma histology and EGFR [corrected] mutation. Journal of thoracic oncology.

[42] Shen M, Rothman N, Berndt SI, He X, Yeager M, Welch R, Chanock S, Caporaso N, Lan Q (2005). Polymorphisms in folate metabolic genes and lung cancer risk in Xuan Wei, China. Lung cancer (Amsterdam, Netherlands).

[43] Herbig K, Chiang EP, Lee LR, Hills J, Shane B, Stover PJ (2002). Cytoplasmic serine hydroxymethyltransferase mediates competition between folate-dependent deoxyribonucleotide and S-adenosylmethionine biosyntheses. The Journal of biological chemistry.

[44] Wang L, Lu J, An J, Shi Q, Spitz MR, Wei Q (2007). Polymorphisms of cytosolic serine hydroxymethyltransferase and risk of lung cancer:a case-control analysis. Lung cancer (Amsterdam, Netherlands).

[45] Swartz MD, Peterson CB, Lupo PJ, Wu X, Forman MR, Spitz MR, Hernandez LM, Vannucci M, Shete S (2013). Investigating multiple candidate genes and nutrients in the folate metabolism pathway to detect genetic and nutritional risk factors for lung cancer. PloS one.

[46] Ulrich CM, Reed MC, Nijhout HF (2008). Modeling folate, one-carbon metabolism, and DNA methylation. Nutrition reviews.

[47] Matakidou A, El Galta R, Rudd MF, Webb EL, Bridle H, Eisen T, Houlston RS (2007). Prognostic significance of folate metabolism polymorphisms for lung cancer. British journal of cancer.

[48] Paone A, Marani M, Fiascarelli A, Rinaldo S, Giardina G, Contestabile R, Paiardini A, Cutruzzola F (2014). SHMT1 knockdown induces apoptosis in lung cancer cells by causing uracil misincorporation. Cell death & disease.

[49] Liu J, Schmitz JC, Lin X, Tai N, Yan W, Farrell M, Bailly M, Chen T, Chu E (2002). Thymidylate synthase as a translational regulator of cellular gene expression. Biochimica et biophysica acta.

[50] Shi Q, Zhang Z, Neumann AS, Li G, Spitz MR, Wei Q (2005). Case-control analysis of thymidylate synthase polymorphisms and risk of lung cancer. Carcinogenesis.

[51] Li HX, Lei DS, Wang XQ, Skog S, He Q (2005). Serum thymidine kinase 1 is a prognostic and monitoring factor in patients with non-small cell lung cancer. Oncology reports.

[52] Greengard O, Head JF, Goldberg SL (1980). Uridine kinase, adenylate kinase, and guanase in human lung tumors. Cancer research.

[53] D‘Angiolella V, Donato V, Forrester FM, Jeong YT, Pellacani C, Kudo Y, Saraf A, Florens L, Washburn MP, Pagano M (2012). Cyclin F-mediated degradation of ribonucleotide reductase M2 controls genome integrity and DNA repair. Cell.

[54] Zhang YW, Jones TL, Martin SE, Caplen NJ, Pommier Y (2009). Implication of checkpoint kinase-dependent up-regulation of ribonucleotide reductase R2 in DNA damage response. The Journal of biological chemistry.

[55] Rahman MA, Amin AR, Wang D, Koenig L, Nannapaneni S, Chen Z, Wang Z, Sica G, Deng X, Chen ZG, Shin DM (2013). RRM2 regulates Bcl-2 in head and neck and lung cancers: a potential target for cancer therapy. Clinical cancer research.

[56] Lee JK, Kim KC (2013). DZNep, inhibitor of S-adenosylhomocysteine hydrolase, down-regulates expression of SETDB1 H3K9me3 HMTase in human lung cancer cells. Biochemical and biophysical research communications.

[57] Gautschi O (2010). The polyamine metabolism: renaissance of an old pathway in oncology. Clinical lung cancer.

[58] Grimminger PP, Schneider PM, Metzger R, Vallbohmer D, Danenberg KD, Danenberg PV, Holscher AH, Brabender J (2010). Ornithine decarboxylase mRNA expression in curatively resected non-small-cell lung cancer. Clinical lung cancer.

[59] Gerner EW (2010). Cancer chemoprevention locks onto a new polyamine metabolic target. Cancer prevention research (Philadelphia, Pa).

[60] Shah N, Thomas TJ, Lewis JS, Klinge CM, Shirahata A, Gelinas C, Thomas T (2001). Regulation of estrogenic and nuclear factor kappa B functions by polyamines and their role in polyamine analog-induced apoptosis of breast cancer cells. Oncogene.

[61] van Riggelen J, Yetil A, Felsher DW (2010). MYC as a regulator of ribosome biogenesis and protein synthesis. Nat Rev Cancer.

[62] Wu M, Tu T, Huang Y, Cao Y (2013). Suppression subtractive hybridization identified differentially expressed genes in lung adenocarcinoma: ERGIC3 as a novel lung cancer-related gene. BMC cancer.

[63] Koga M, Shichijo S, Yamada A, Ashihara J, Sawamizu H, Kusukawa J, Itoh K (2003). Identification of ribosomal proteins S2 and L10a as tumor antigens recognized by HLA-A26-restricted CTL. Tissue antigens.

[64] Russo N, Wang X, Liu M, Banerjee R, Goto M, Scanlon C, Metwally T, Inglehart RC, Tsodikov A, Duffy S, Van Tubergen E, Bradford C, Carey T (2013). A novel approach to biomarker discovery in head and neck cancer using an autoantibody signature. Oncogene.

[65] Kim JH, You KR, Kim IH, Cho BH, Kim CY, Kim DG (2004). Over-expression of the ribosomal protein L36a gene is associated with cellular proliferation in hepatocellular carcinoma. Hepatology.

[66] Cowling VH, Turner SA, Cole MD (2014). Burkitt‘s lymphoma-associated c-Myc mutations converge on a dramatically altered target gene response and implicate Nol5a/Nop56 in oncogenesis. Oncogene.

[67] Mei YP, Liao JP, Shen J, Yu L, Liu BL, Liu L, Li RY, Ji L, Dorsey SG, Jiang ZR, Katz RL, Wang JY, Jiang F (2012). Small nucleolar RNA 42 acts as an oncogene in lung tumorigenesis. Oncogene.

[68] Renieri A, Mencarelli MA, Cetta F, Baldassarri M, Mari F, Furini S, Piu P, Ariani F, Dragani TA, Frullanti E (2014). Oligogenic germline mutations identified in early non-smokers lung adenocarcinoma patients. Lung cancer (Amsterdam, Netherlands).

[69] Grisendi S, Mecucci C, Falini B, Pandolfi PP (2006). Nucleophosmin and cancer. Nat Rev Cancer.

[70] Mendes-da-Silva P, Moreira A, Duro-da-Costa J, Matias D, Monteiro C (2000). Frequent loss of heterozygosity on chromosome 5 in non-small cell lung carcinoma. Molecular pathology.

[71] Pang Q, Christianson TA, Koretsky T, Carlson H, David L, Keeble W, Faulkner GR, Speckhart A, Bagby GC (2003). Nucleophosmin interacts with and inhibits the catalytic function of eukaryotic initiation factor 2 kinase PKR. The Journal of biological chemistry.

[72] Greasley PJ, Bonnard C, Amati B (2000). Myc induces the nucleolin and BN51 genes:possible implications in ribosome biogenesis. Nucleic acids research.

[73] Ding Y, Song N, Liu C, He T, Zhuo W, He X, Chen Y, Song X, Fu Y, Luo Y (2012). Heat shock cognate 70 regulates the translocation and angiogenic function of nucleolin. Arteriosclerosis, Thrombosis, and Vascular Biology.

[74] Tsou JH, Chang KY, Wang WC, Tseng JT, Su WC, Hung LY, Chang WC, Chen BK (2008). Nucleolin regulates c-Jun/Sp1-dependent transcriptional activation of cPLA2alpha in phorbol ester-treated non-small cell lung cancer A549 cells. Nucleic acids research.

[75] Wang D, Xiang DB, Yang XQ, Chen LS, Li MX, Zhong ZY, Zhang YS (2009). APE1 overexpression is associated with cisplatin resistance in non-small cell lung cancer and targeted inhibition of APE1 enhances the activity of cisplatin in A549 cells. Lung cancer (Amsterdam, Netherlands).

[76] Seo SB, Macfarlan T, McNamara P, Hong R, Mukai Y, Heo S, Chakravarti D (2002). Regulation of histone acetylation and transcription by nuclear protein pp32, a subunit of the INHAT complex. The Journal of biological chemistry.

[77] Schramedei K, Morbt N, Pfeifer G, Lauter J, Rosolowski M, Tomm JM, von Bergen M, Horn F, Brocke-Heidrich K (2011). MicroRNA-21 targets tumor suppressor genes ANP32A and SMARCA4. Oncogene.

[78] Shen F, Look KY, Yeh YA, Weber G (1998). Increased uridine kinase (ATP: uridine 5′-phosphotransferase; EC 2.7.1.48) activity in human and rat tumors. Cancer biochemistry biophysics.

[79] Goel A, Mathupala SP, Pedersen PL (2003). Glucose metabolism in cancer. Evidence that demethylation events play a role in activating type II hexokinase gene expression. The Journal of biological chemistry.

[80] Natsumeda Y, Ohno S, Kawasaki H, Konno Y, Weber G, Suzuki K (1990). Two distinct cDNAs for human IMP dehydrogenase. The Journal of biological chemistry.

[81] Wikman H, Kettunen E, Seppanen JK, Karjalainen A, Hollmen J, Anttila S, Knuutila S (2002). Identification of differentially expressed genes in pulmonary adenocarcinoma by using cDNA array. Oncogene.

[82] Suer Gokmen S, Yoruk Y, Cakir E, Yorulmaz F, Gulen S (1999). Arginase and ornithine, as markers in human non-small cell lung carcinoma. Cancer biochemistry biophysics.

[83] Nakagawa T, Otake Y, Yanagihara K, Miyahara R, Ishikawa S, Fukushima M, Wada H, Tanaka F (2004). Expression of thymidylate synthase is correlated with proliferative activity in non-small cell lung cancer (NSCLC). Lung cancer (Amsterdam, Netherlands).

[84] Rotenberg Z, Weinberger I, Sagie A, Fuchs J, Davidson E, Sperling O, Agmon J (1988). Total lactate dehydrogenase and its isoenzymes in serum of patients with non-small-cell lung cancer. Clinical chemistry.

[85] Temme A, Rieger M, Reber F, Lindemann D, Weigle B, Diestelkoetter-Bachert P, Ehninger G, Tatsuka M, Terada Y, Rieber EP (2003). Localization, dynamics, and function of survivin revealed by expression of functional survivinDsRed fusion proteins in the living cell. Molecular biology of the cell.

[86] Bisio A, Zamborszky J, Zaccara S, Lion M, Tebaldi T, Sharma V, Raimondi I, Alessandrini F, Ciribilli Y, Inga A (2014). Cooperative interactions between p53 and NFkappaB enhance cell plasticity. Oncotarget.

[87] Gyorffy B, Surowiak P, Budczies J, Lanczky A (2013). Online survival analysis software to assess the prognostic value of biomarkers using transcriptomic data in non-small-cell lung cancer. PloS one.

[88] Huang B, Zhou H, Wang X, Liu Z (2013). Silencing SATB1 with siRNA inhibits the proliferation and invasion of small cell lung cancer cells. Cancer cell international.

[89] Selinger CI, Cooper WA, Al-Sohaily S, Mladenova DN, Pangon L, Kennedy CW, McCaughan BC, Stirzaker C, Kohonen-Corish MR (2011). Loss of special AT-rich binding protein 1 expression is a marker of poor survival in lung cancer. Journal of thoracic oncology.

