# Supplementary material for: c-Myc targeted regulators of cell metabolism in a transgenic mouse model of papillary lung adenocarcinoma
Source: Oncotarget. 2016 Sep 1;7(40):65514–39. doi: 10.18632/oncotarget.11804 (PMC5323172; doi:10.18632/oncotarget.11804)
Supplement: Supplementary file 1 [file oncotarget-07-65514-s001.pdf]

c-MYC targeted regulators of cell metabolism in a transgenic mouse model of papillary lung adenocarcinoma

Supplementary Materials

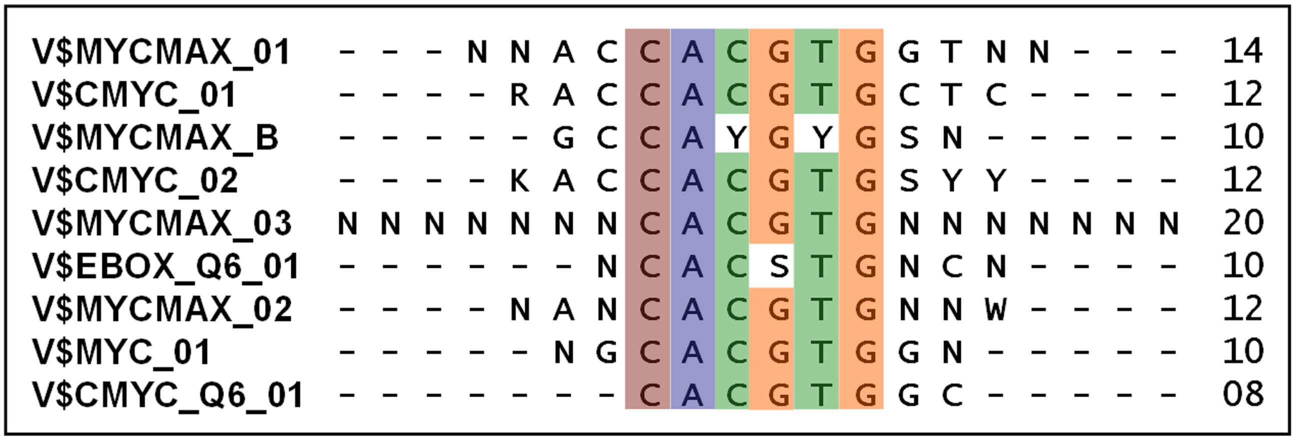

Supplementary Figure S1: Consensus sequence alignment of 9 different position weight matrices of c-Myc. Multiple sequence alignment with nine different PWM for c-Myc was done using the Clustal-W2 software (http://www.ebi.ac.uk/Tools/msa/clustalw2/); the consensus sequences were taken from TRANSFAC(R) database.

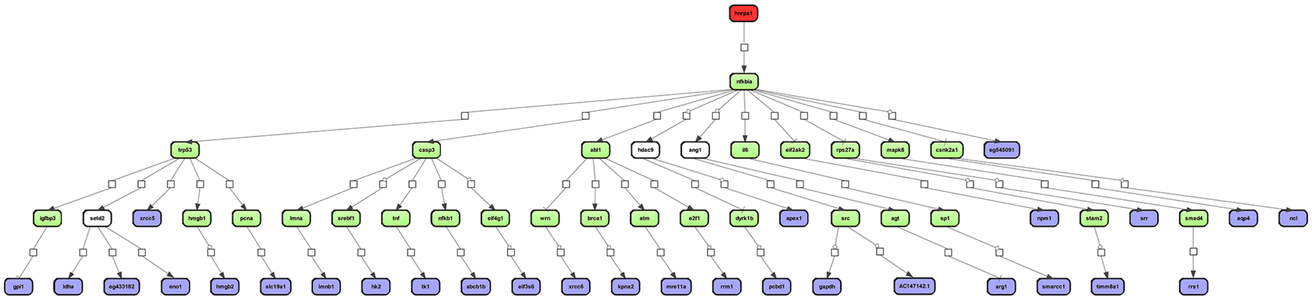

Supplementary Figures S2: Master regulatory networks of PLAC regulated genes. The networks for significant master regulatory gene networks were constructed with the GeneXplain platform. An activation, inactivation and regulation is denoted by the symbols, respectively. S2 to S8 refers to the *Hnrpa1 Apex1 Tpi1 Slc19a1 Ncl, Npm1* and *Gapdh* master regulatory gene networks, respectively.

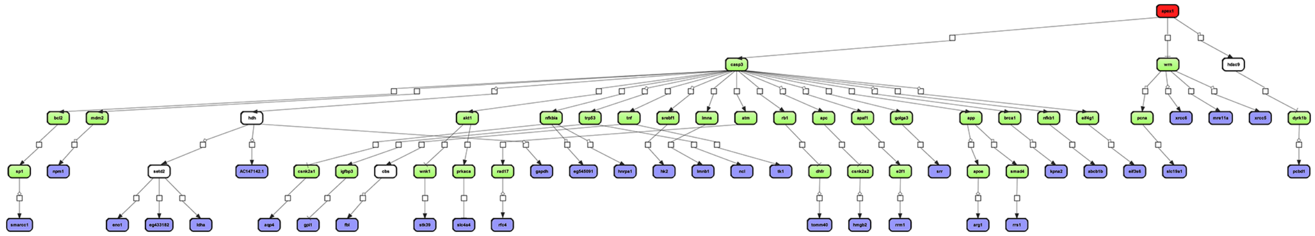

Supplementary Figures S3: Master regulatory networks of PLAC regulated genes. The networks for significant master regulatory gene networks were constructed with the GeneXplain platform. An activation, inactivation and regulation is denoted by the symbols , respectively. S2 to S8 refers to the *Hnrpa1 Apex1 Tpi1 Slc19a1 Ncl, Npm1* and *Gapdh* master regulatory gene networks, respectively.

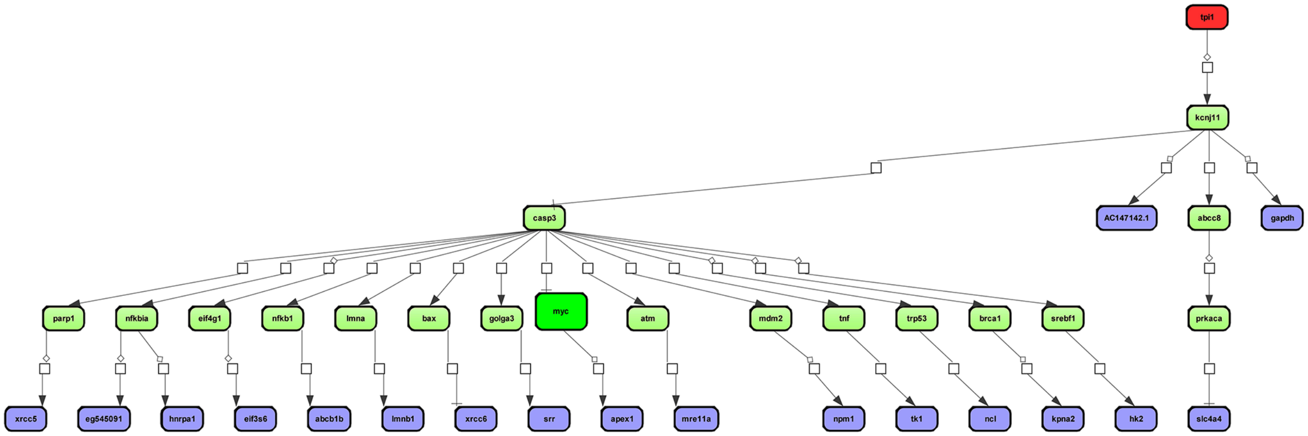

Supplementary Figures S4: Master regulatory networks of PLAC regulated genes. The networks for significant master regulatory gene networks were constructed with the GeneXplain platform. An activation, inactivation and regulation is denoted by the symbols , respectively. S2 to S8 refers to the *Hnrpa1 Apex1 Tpi1 Slc19a1 Ncl, Npm1* and *Gapdh* master regulatory gene networks, respectively.

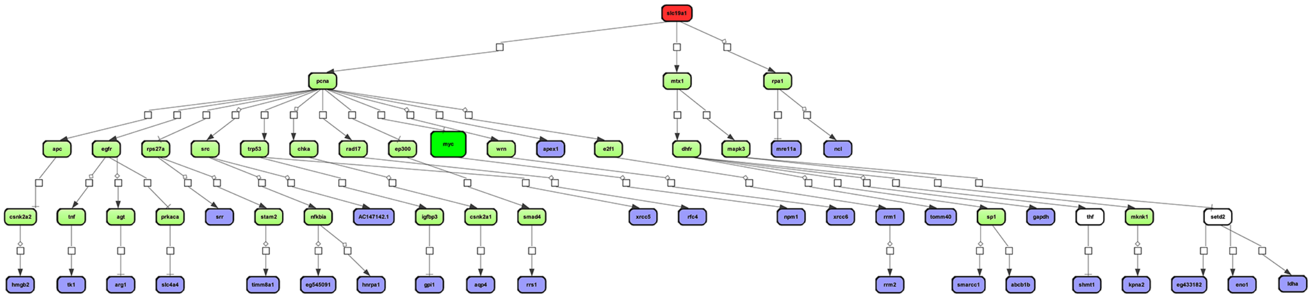

Supplementary Figures S5: Master regulatory networks of PLAC regulated genes. The networks for significant master regulatory gene networks were constructed with the GeneXplain platform. An activation, inactivation and regulation is denoted by the symbols , respectively. S2 to S8 refers to the *Hnrpa1 Apex1 Tpi1 Slc19a1 Ncl, Npm1* and *Gapdh* master regulatory gene networks, respectively.

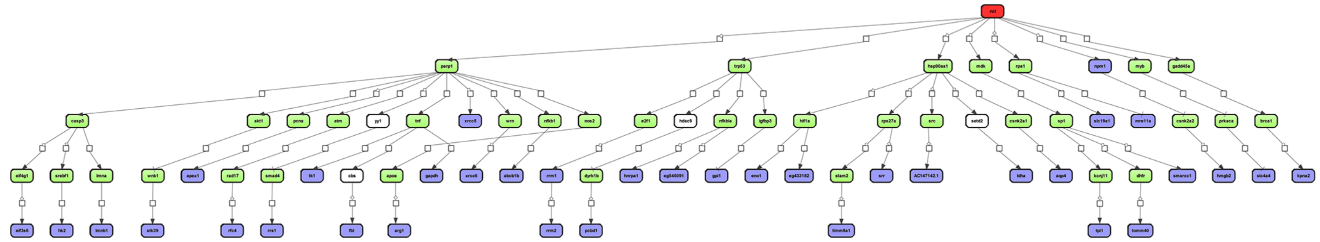

Supplementary Figures S6: Master regulatory networks of PLAC regulated genes. The networks for significant master regulatory gene networks were constructed with the GeneXplain platform. An activation, inactivation and regulation is denoted by the symbols , respectively. S2 to S8 refers to the *Hnrpa1 Apex1 Tpi1 Slc19a1 Ncl, Npm1* and *Gapdh* master regulatory gene networks, respectively.

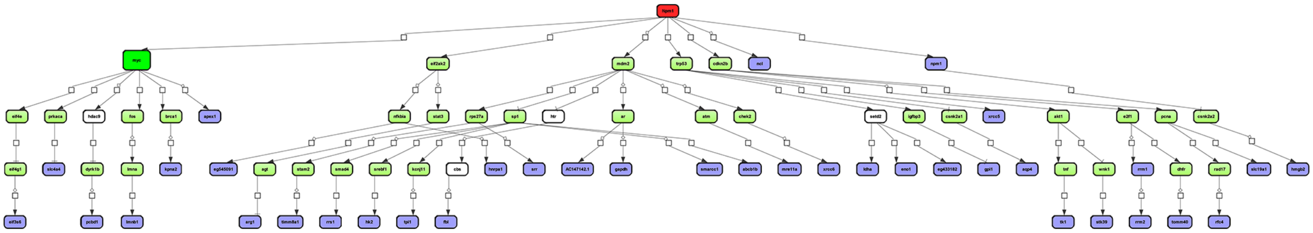

Supplementary Figures S7: Master regulatory networks of PLAC regulated genes. The networks for significant master regulatory gene networks were constructed with the GeneXplain platform. An activation, inactivation and regulation is denoted by the symbols , respectively. S2 to S8 refers to the *Hnrpa1 Apex1 Tpi1 Slc19a1 Ncl, Npm1* and *Gapdh* master regulatory gene networks, respectively.

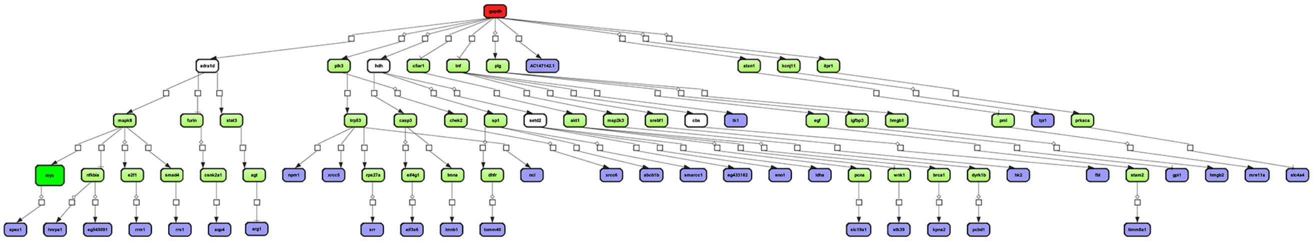

Supplementary Figures S8: Master regulatory networks of PLAC regulated genes. The networks for significant master regulatory gene networks were constructed with the GeneXplain platform. An activation, inactivation and regulation is denoted by the symbols , respectively. S2 to S8 refers to the *Hnrpa1 Apex1 Tpi1 Slc19a1 Ncl, Npm1* and *Gapdh* master regulatory gene networks, respectively.

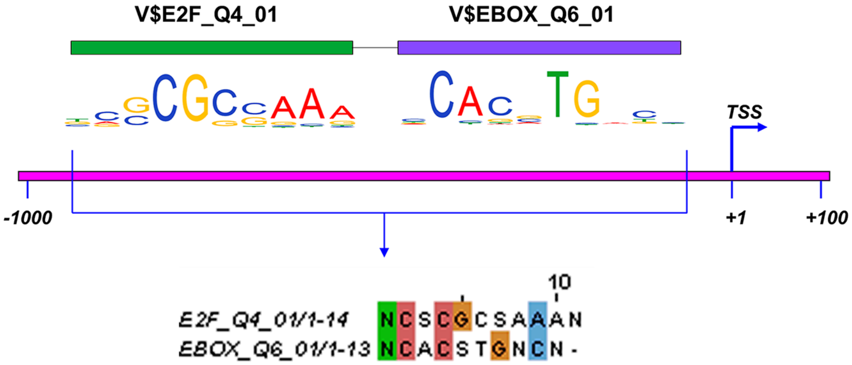

Supplementary Figure S9: Co-occupancy of transcription factor binding sites (composite module) in tumor associated gene regulations. The co-occupancy of E2F and EBox transcription factor binding sites at gene specific promoter is depicted. The software Clustal Omega (http://www.ebi.ac.uk/Tools/msa/clustalo/) was used for multiple sequence alignment (MSA) to define overlapping regions. The E2F, EBox and the composite module fitted 71%, 79% and 67%, respectively of the up-regulated genes in PLACs of c-MYC transgenic mice (see Supplementary Table S10).

Supplementary Table S1: c-MYC binding sites in PLAC regulated genes. see Supplementary_Table_S1

Supplementary Table S2: Frequency of c-Myc binding motifs in PLAC regulated genes. see Supplementary_Table_S2

Supplementary Table S3: Master regulatory gene networks. see Supplementary_Table_S3

Supplementary Table S4: Composite module construction for PLAC regulated genes

Result Values							
**Regulated Data**	**Modules**	**Model1**	**Model2**	**Module width**	**Score of the best match and number of individual matches for each PWM in CM's Models**		**CM Score**
					**Model1**	**Model2**	
**Up-regulated Tumor**	Module1	V$E2F_Q4_01	V$EBOX_Q6_01	73	0.78; *N* = 2	0.94; *N* = 2	27.68

Value of Parameters During CM Search										
**Regulated Data**	**Number of iterations**	**Population size**	**Non-change limit**	**Elite size**	**Mutation Rate**	**Penalty rate**	**Modules**	**Models**	**Sites to account**	**Module width**
**Up-regulated Tumor**	300	1000	300	100	0,7	0.3	1	2	1 to 3	5 to 100

Supplementary Table S5: Co-occupancy of c-Myc and TFDP1 transcription factor binding sites in PLAC regulated genes. See Supplementary_Table_S5

Supplementary Table S6: Sequence information for EMSA probes

Mouse_Shmt1_BS 2_F	5′-TGGTGTGTCACGTGGTGGGGAGG
Mouse_Hk1_BS 1_F	5′-CTCTTCTACCACGTGGGTTCTGG
Mouse_Gapd_BS 1_F	5′-GGAACAACCCCACGCGCCCGTTC
Mouse_Tpi_BS 1_F	5′-AGCAAGTGGCACGTGTCCCCCAG
Mouse_Gart_BS 1_F	5′-CGAAAAGCCCACGTGCTTTGCAC
Mouse_Impdh2_BS 1_F	5′-CTCGATAAGCACGTGGTTACCCA
Mouse_Uck2-pending_BS 1_F	5′-CTGCCACCGCGTGGCCCGCGGGA
Mouse_Apex1_BS 1_F	5′-GAACGAACCCACGTGACCTAGCC
Mouse_Smarcc1_BS 1_F	5′-ACCGAGGAGCCACGCGACGGGGG
Mouse_Nol5a_BS 1_F	5′-ATGAGCAACCACGTGGTGCCGAG
Mouse_Ncl_BS 2_F	5′-GCGGCCCGCCACGTGCTCTGCGG
Mouse_Npm1_BS 2_F	5′-GTGGTTGCCACGTGGTTGGGGGT
Mouse_Npm3_BS 2_F	5′-GACACGGAGCACGTGCTGGCGTT
Mouse_Hnrpa1_BS 1_F	5′-CGCCATTTCACGTGTTCCAGGCA
Mouse_Fbl_BS 1_F	5′-CGTCTTGTCACGTGATCCTAGCT
Mouse_Pabpc4_BS 1_F	5′-TCTGCGCCGCGTGGCAGCCCCGC
Mouse_Nola1_BS 1_F	5′-GGAGACGCACGTGGCGCGGTGAG
Mouse_Ppan_BS 1_F	5′-TACGAGCCGCGTGGCACCGGAAG
Mouse_Rnac-pending_BS 3_F	5′-CGAGCTGCGCCACGCGTGGAAGG
Mouse_Rpl27a_BS 1_F	5′-ACTCCGGGGCCACGTGAGCCACG
Mouse_Bzw2_BS 2_F	5′-GTAAGCTGGCCACGCGGTGAAAG
Mouse_Eif3s6_BS 1_F	5′-GAGAGGCCACCACGCGCAACTGC
Mouse_Cct5_BS 1_F	5′-GCACCGCAGCCACGCGCGCCCGA
Mouse_Fkbp11_BS 1_F	5′-GCTTGGACACGTGGCAAGCTATC
Mouse_Pycs_BS 1_F	5′-AGGGGGCCGCCACGTGGAGCGTG
Mouse_Lamr1_BS 2_F	5′-CGGCAGCGCCACGTGCTGGCCCG
Mouse_Timm10_BS 1_F	5′-ACATTTTCCCACGTGGTAACGAA
Mouse_Timm8a_BS 1_F	5′-TCAGTGGACCACGTGGGATGAAG
Mouse_Timm8a_BS 2_F	5′-CTCGGCCCGCGTGGTCGTGCCGG
Mouse_Slc19a1_BS 1_F	5′-ACTTTAGAGCACGTGTTGCCTGC
Mouse_Rangnrf-pending_BS 1_F	5′-ACTCTGAGGCACGTGACCCTTGC
Mouse_Mlp_BS 1_F	5′-GGAGAATGGCCACGTGAGAAGCA
Mouse_Abcb1b_BS 1_F	5′-CCCCGCGGCCACGTGTTTGGCTA
c-Myc_positive control_BS_WT_F_R	5′-GGAAGCAGACCACGTGGTCTGCTTCC
c-Myc_positive control_BS_Mut_F	5′-GGAAGCAGACCACGGAGTCTGCTTCC
c-Myc_positive control_BS_Mut_F	5′-GGAAGCAGACTCCGTGGTCTGCTTCC

Supplementary Table S7: ChiP-seq data retrieved from public repositories.
See Supplementary_Table_S7

Supplementary Table S8: Comparison EMSA data of the current study with published Ref-seq/ Chip-seq data. See Supplementary_Table_S8

Supplementary Table S9: Hazard ratios for individual PLAC regulated genes.
See Supplementary_Table_S9

Supplementary Table S10: Search for statistically significantly regulated transcription factor binding sites in PLAC regulated genes

ID	Yes density per 1000 bp	No density per 1000 bp	Yes-No ratio	*P*-value
V$CMYC_Q6_01	1,71368861	0,016029311	106,909688	1,10E-111
V$MYCMAX_02	1,003134796	0,006869705	146,0229885	2,45E-67
V$MYC_01	0,65830721	0,004579803	143,7413793	1,56E-44
V$CMYC_01	0,595611285	0,002289902	260,1034483	1,57E-41
V$MYCMAX_01	0,585161964	0,004579803	127,7701149	2,05E-39
V$CMYC_02	0,480668757	0,004579803	104,954023	3,97E-32
V$E2F_Q4_01	6,175548589	3,476070529	1,776588978	6,65E-30
V$E2F_Q4_02	6,029258098	3,439432104	1,7529807	4,08E-28
V$E2F_Q3_01	5,548589342	3,128005496	1,773842581	6,18E-27
V$E2F_Q2	4,639498433	2,564689718	1,808990148	4,11E-24
V$E2F_Q6_01	4,190177638	2,262422716	1,85207548	2,20E-23
V$KLF7_04	2,946708464	1,722005954	1,711206897	1,13E-13
V$P53_01	3,94984326	2,802839478	1,409229209	9,23E-09
V$P53_03	3,260188088	2,26929242	1,436654024	3,84E-08
V$P53_04	1,421107628	0,858713075	1,654927203	8,61E-07
V$SMAD6_01	0,39707419	0,139683994	2,842660637	1,42E-06
V$KLF7_03	0,679205852	0,345775132	1,964299307	1,00E-05
V$KLF7_06	0,480668757	0,242729563	1,980264585	1,57E-04
V$AREB6_02	0,146290491	0,036638425	3,992816092	2,90E-04
V$GKLF_02	0,365726228	0,171742615	2,129501916	3,20E-04

Supplementary Table S11: Biological pathways mapping over PPI-networks

Selection criteria for functional grouping of pathway terms

Selection citeria for pathway terms	Statistical Test Used	Enrichment (Right-sided hypergeometric test)
	Correction Method Used	Bonferroni step down
	Min GO Level	3
	Max GO Level	8
	Minimum number of Genes or Proteins	2
	Min percentage of Genes or Proteins	1
Selection citeria for grouping of pathway terms	GO Group	true
	Kappa Score Threshold	0.4
	Over View Term	Smallest *P* Value
	Group By Kappa Statistics	true
	Initial Group Size	2
	Sharing Group Percentage	50

Supplementary Table S12: Primer pairs for gene expression studies by RT-PCR

Gene symbol	Forward primer	Reverse primer	annealing temperatur	cycles
**Shmt1**	GCAACTCTGAACCAGTGCAA	TGAGGATCCAGATGGTAGGC (, 36 cycles)	56°C	36
**Satb1**	GTGATGGCTCAGTTGCTGAA	CATAGCCCGAAGGTTTACCA (, 32 cycles)	57°C	32
**Arg1**	AAGCTGGTCTGCTGGAAAAA	CTGGTTGTCAGGGGAGTGTT (, 36 cycles)	55°C	36
**Fasn**	TCTGCAGAGAAGCGAGCATA	GTCATTGGCCTCCTCAAAAA (, 31 cycles)	55°C	31
**Srm**	GTCCAGTGCGAGATTGATGA	GCAGAAGTGCCTCATCTCCT (, 32 cycles)	57°C	32
**Hk2**	GGTGGAGATGGAGAACCAGA	TCATTCACCACAGCCACAAT (, 32 cycles)	55°C	32
**Tk1**	CCAGATCGCCCAGTACAAGT	GAAGCACTCCATGCACACAG (, 34 cycles)	59°C	34
**Impdh2**	GGAAGTGGTTCCATCTGCAT	TGGCTGCTGAGATGTTTGTC (, 32 cycles)	57°C	32
**Uck2**	CAGATCCCCGTGTACGACTT	GTCGGCACCTCTAGGAATGA (, 32 cycles)	59°C	32
**Smarcc1**	TGATCCAAGTCGCTCAGTTG	TCACAGCACACACATCTCCA (, 32 cycles)	57°C	32
**Npm3**	GGCTGCTTTAGCGTTCTTGA	AAGGTGACAGGTGGTTGGAG (, 33 cycles)	57°C	33
**Slc19a1**	ATGTGCATGTCCTGTGGAGA	AGGGAAGACGCAATCTGAAA (, 37 cycles)	55°C	37
**ß-actin, housekeeping gene**	GGCATTGTTACCAACTGGGACG	CTCTTTGATGTCACGCACGATTTC (, 23 cycles)	65°C	23

Supplementary Table S13: Primer sequences

ChIP qPCR primers	SEQUENCES
**ACTB-Fw**	TCGCGCCGCTGGGTTTTATA
**ACTB-Rv**	TCTCCCTCCTCCTCTTCCTCAAT
**CCNB1_ex9-Fw**	TATGCCACATCGAAGCATGCTAA
**CCNB1_ex9-Rv**	ACAGATGGCACATGGTGCCAATT
**CCND2-Fw**	AGTGAAATACACCAAAGGGCG
**CCND2-Rv**	CTCACTCGCCAGGCTTTCT
**CDK4-Fw**	ATGTGACCAGCTGCCAAAG
**CDK4-Rv**	CCTCTGCTCCTCAGAGCAAT
**HK1-Fw**	CATTGGACCAGGGAGACAGT
**HK1-Rv**	GGTGTGGACACTGGAGGTTT
**NPM3-Fw**	AGACGGAGGTGGACGTGTAA
**NPM3-Rv**	CGGAGGTTGGGTAGAGACCT
**RCL1-Fw**	TCCGTCTTCCTGTTCCAAAC
**RCL1-Rv**	GCTCTCCGAAGAGACGTGAG
**RPSA-Fw**	GTTCTGGGTGAGTTCCGTGT
**RPSA-Rv**	AGGATGTTAGCCCGCTTTCT
**RT-qPCR primers**	**SEQUENCES**
**GAPDH-Fw**	GAAGGTGAAGGTCGGAGTC
**GAPDH-Rv**	GAAGATGGTGATGGGATTTC
**YWHAZ-Fw**	ACTTTTGGTACTTTGTGGCTTCAA
**YWHAZ-Rv**	CCGCCAAGGGACAAACCAGTAT
**c-MYC-Fw**	GCCACGTCTCCACACATCAG
**c-MYC-Rv**	TGGTGCATTTTCGGTTGTTG
