# Supplementary figures and images for: c-Myc targeted regulators of cell metabolism in a transgenic mouse model of papillary lung adenocarcinoma
Source: Oncotarget. 2016 Sep 1;7(40):65514–39. doi: 10.18632/oncotarget.11804 (PMC5323172; doi:10.18632/oncotarget.11804)

**Supplementary Table S1: c-MYC binding sites in PLAC regulated genes**


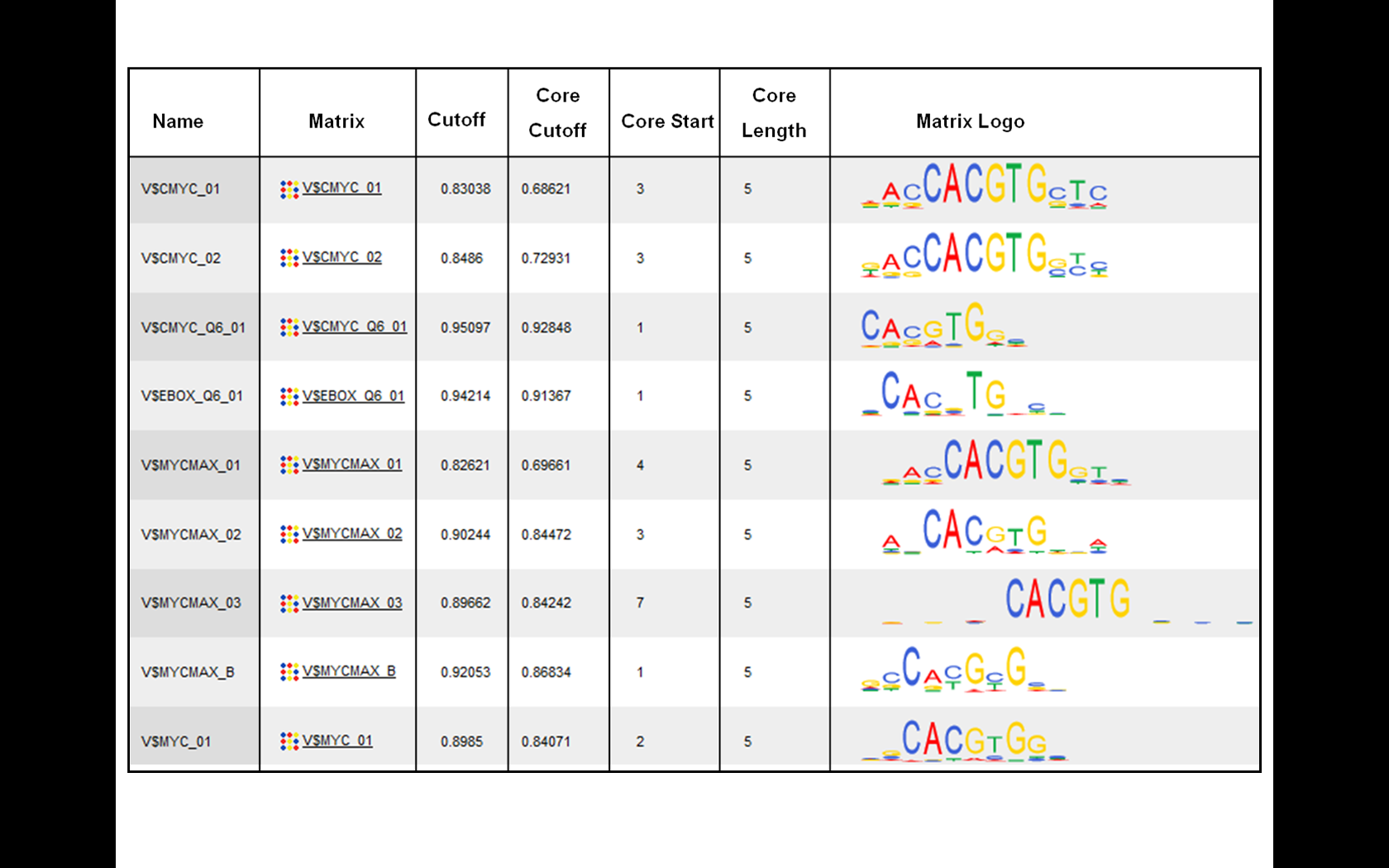

Supplement: Supplementary file 2 [file oncotarget-07-65514-s002.docx]
