# Supplementary material for: c-Myc targeted regulators of cell metabolism in a transgenic mouse model of papillary lung adenocarcinoma
Source: Oncotarget. 2016 Sep 1;7(40):65514–39. doi: 10.18632/oncotarget.11804 (PMC5323172; doi:10.18632/oncotarget.11804)
Supplement: Supplementary file 3 [file oncotarget-07-65514-s003.docx]

**Supplementary Table S2**: **Frequency of c-Myc binding motifs in PLAC regulated genes**

| **No.** | **ID** | **Symbol** | **Avarage count (based on total cMyc count /No. of positive PWMs )** | **V$EBOX_Q6_01** | **V$MYCMAX_B** | **V$MYCMAX_02** | **V$MYC_01** | **V$MYCMAX_01** | **V$MYCMAX_03** | **V$CMYC_02** | **V$CMYC_01** | **V$CMYC_Q6_01** |
| --- | --- | --- | --- | --- | --- | --- | --- | --- | --- | --- | --- | --- |
| 1 | ENSMUSG00000035960 | Apex1 | 7 | 10 | 6 | 8 | 5 | 8 | 8 | 7 | 6 | 5 |
| 2 | ENSMUSG00000019987 | Arg1 | 4 | 5 | 0 | 2 | 0 | 0 | 0 | 0 | 0 | 0 |
| 3 | ENSMUSG00000079435 | Rpl36a | 4 | 5 | 0 | 2 | 0 | 0 | 0 | 0 | 0 | 0 |
| 4 | ENSMUSG00000003355 | Fkbp11 | 4 | 4 | 0 | 0 | 0 | 0 | 0 | 0 | 0 | 0 |
| 5 | ENSMUSG00000025574 | Tk1 | 4 | 4 | 0 | 0 | 0 | 0 | 0 | 0 | 0 | 0 |
| 6 | ENSMUSG00000037012 | Hk1 | 3 | 10 | 3 | 5 | 2 | 2 | 2 | 2 | 2 | 2 |
| 7 | ENSMUSG00000024590 | Lmnb1 | 3 | 4 | 4 | 4 | 3 | 4 | 4 | 2 | 1 | 2 |
| 8 | ENSMUSG00000006442 | Srm | 3 | 6 | 3 | 3 | 2 | 2 | 2 | 2 | 1 | 2 |
| 9 | ENSMUSG00000027076 | Timm10 | 3 | 5 | 1 | 3 | 1 | 4 | 2 | 3 | 3 | 1 |
| 10 | ENSMUSG00000027405 | Nop56 | 3 | 2 | 4 | 3 | 4 | 2 | 2 | 2 | 2 | 2 |
| 11 | ENSMUSG00000020534 | Shmt1 | 3 | 4 | 2 | 2 | 0 | 0 | 0 | 0 | 0 | 0 |
| 12 | ENSMUSG00000025153 | Fasn | 3 | 2 | 4 | 0 | 2 | 0 | 0 | 0 | 0 | 0 |
| 13 | ENSMUSG00000024640 | Psat1 | 3 | 1 | 0 | 4 | 0 | 0 | 0 | 0 | 0 | 0 |
| 14 | ENSMUSG00000031388 | Naa10 | 3 | 3 | 2 | 0 | 0 | 0 | 0 | 0 | 0 | 0 |
| 15 | ENSMUSG00000057278 | Snrpg | 3 | 2 | 3 | 0 | 0 | 0 | 0 | 0 | 0 | 0 |
| 16 | ENSMUSG00000021556 | Golm1 | 3 | 3 | 0 | 0 | 0 | 0 | 0 | 0 | 0 | 0 |
| 17 | ENSMUSG00000022471 | Xrcc6 | 3 | 3 | 0 | 0 | 0 | 0 | 0 | 0 | 0 | 0 |
| 18 | ENSMUSG00000025001 | Hells | 3 | 0 | 3 | 0 | 0 | 0 | 0 | 0 | 0 | 0 |
| 19 | ENSMUSG00000063229 | Ldha | 3 | 3 | 0 | 0 | 0 | 0 | 0 | 0 | 0 | 0 |
| 20 | ENSMUSG00000024785 | Rcl1 | 2 | 2 | 3 | 2 | 3 | 2 | 2 | 2 | 2 | 2 |
| 21 | ENSMUSG00000048007 | Timm8a1 | 2 | 2 | 4 | 2 | 2 | 2 | 2 | 2 | 2 | 2 |
| 22 | ENSMUSG00000025747 | Tyms | 2 | 5 | 2 | 3 | 1 | 2 | 2 | 1 | 1 | 1 |
| 23 | ENSMUSG00000026187 | Xrcc5 | 2 | 3 | 2 | 2 | 4 | 2 | 2 | 1 | 1 | 1 |
| 24 | ENSMUSG00000061024 | Rrs1 | 2 | 4 | 1 | 3 | 1 | 2 | 2 | 2 | 2 | 1 |
| 25 | ENSMUSG00000062867 | Impdh2 | 2 | 3 | 3 | 2 | 1 | 2 | 2 | 2 | 2 | 1 |
| 26 | ENSMUSG00000021733 | Slc4a7 | 2 | 2 | 1 | 3 | 2 | 2 | 2 | 2 | 2 | 1 |
| 27 | ENSMUSG00000025007 | Aldh18a1 | 2 | 2 | 2 | 2 | 2 | 2 | 2 | 2 | 1 | 2 |
| 28 | ENSMUSG00000020547 | Bzw2 | 2 | 3 | 3 | 2 | 2 | 2 | 2 | 1 | 1 | 0 |
| 29 | ENSMUSG00000032892 | Rangrf | 2 | 4 | 2 | 2 | 1 | 2 | 2 | 2 | 0 | 1 |
| 30 | ENSMUSG00000032518 | Rpsa | 2 | 2 | 2 | 2 | 1 | 2 | 2 | 0 | 0 | 1 |
| 31 | ENSMUSG00000046434 | Hnrnpa1 | 2 | 3 | 0 | 2 | 0 | 2 | 2 | 0 | 1 | 0 |
| 32 | ENSMUSG00000029642 | Polr1d | 2 | 5 | 2 | 0 | 1 | 0 | 0 | 0 | 0 | 1 |
| 33 | ENSMUSG00000000628 | Hk2 | 2 | 4 | 1 | 1 | 0 | 0 | 0 | 0 | 0 | 0 |
| 34 | ENSMUSG00000021474 | Sfxn1 | 2 | 2 | 1 | 1 | 0 | 2 | 0 | 0 | 0 | 0 |
| 35 | ENSMUSG00000036427 | Gpi1 | 2 | 3 | 1 | 1 | 0 | 0 | 0 | 0 | 1 | 0 |
| 36 | ENSMUSG00000029388 | Eif2b1 | 2 | 2 | 2 | 1 | 0 | 0 | 0 | 0 | 0 | 0 |
| 37 | ENSMUSG00000044927 | H1fx | 2 | 2 | 2 | 0 | 1 | 0 | 0 | 0 | 0 | 0 |
| 38 | ENSMUSG00000011257 | Pabpc4 | 2 | 3 | 1 | 0 | 0 | 0 | 0 | 0 | 0 | 0 |
| 39 | ENSMUSG00000026558 | Uck2 | 2 | 2 | 2 | 0 | 0 | 0 | 0 | 0 | 0 | 0 |
| 40 | ENSMUSG00000027374 | Mrps5 | 2 | 2 | 2 | 0 | 0 | 0 | 0 | 0 | 0 | 0 |
| 41 | ENSMUSG00000057113 | Npm1 | 2 | 2 | 2 | 0 | 0 | 0 | 0 | 0 | 0 | 0 |
| 42 | ENSMUSG00000061838 | Suclg2 | 2 | 2 | 2 | 0 | 0 | 0 | 0 | 0 | 0 | 0 |
| 43 | ENSMUSG00000022899 | Slc15a2 | 2 | 2 | 0 | 1 | 0 | 0 | 0 | 0 | 0 | 0 |
| 44 | ENSMUSG00000024411 | Aqp4 | 2 | 2 | 0 | 1 | 0 | 0 | 0 | 0 | 0 | 0 |
| 45 | ENSMUSG00000026020 | Nop58 | 2 | 0 | 0 | 0 | 0 | 2 | 0 | 1 | 0 | 0 |
| 46 | ENSMUSG00000039640 | Mrpl12 | 2 | 2 | 0 | 0 | 1 | 0 | 0 | 0 | 0 | 0 |
| 47 | ENSMUSG00000065087 | Snord22 | 2 | 2 | 0 | 1 | 0 | 0 | 0 | 0 | 0 | 0 |
| 48 | ENSMUSG00000001436 | Slc19a1 | 2 | 2 | 0 | 0 | 0 | 0 | 0 | 0 | 0 | 0 |
| 49 | ENSMUSG00000002319 | Ipo4 | 2 | 2 | 0 | 0 | 0 | 0 | 0 | 0 | 0 | 0 |
| 50 | ENSMUSG00000023456 | Tpi1 | 2 | 2 | 0 | 0 | 0 | 0 | 0 | 0 | 0 | 0 |
| 51 | ENSMUSG00000057666 | Gapdh | 2 | 2 | 0 | 0 | 0 | 0 | 0 | 0 | 0 | 0 |
| 52 | ENSMUSG00000074129 | Rpl13a | 2 | 2 | 0 | 0 | 0 | 0 | 0 | 0 | 0 | 0 |
| 53 | ENSMUSG00000046865 | Fbl | 1 | 2 | 1 | 2 | 1 | 2 | 2 | 1 | 1 | 1 |
| 54 | ENSMUSG00000037805 | Rpl10a | 1 | 2 | 1 | 1 | 1 | 0 | 0 | 0 | 0 | 0 |
| 55 | ENSMUSG00000004100 | Ppan | 1 | 1 | 1 | 0 | 2 | 0 | 0 | 0 | 0 | 0 |
| 56 | ENSMUSG00000026234 | Ncl | 1 | 2 | 1 | 0 | 1 | 0 | 0 | 0 | 0 | 0 |
| 57 | ENSMUSG00000026377 | Mki67ip | 1 | 1 | 1 | 1 | 1 | 0 | 0 | 0 | 0 | 0 |
| 58 | ENSMUSG00000031754 | Nudt21 | 1 | 2 | 0 | 1 | 0 | 0 | 0 | 0 | 0 | 1 |
| 59 | ENSMUSG00000037722 | Gnpnat1 | 1 | 1 | 1 | 0 | 2 | 0 | 0 | 0 | 0 | 0 |
| 60 | ENSMUSG00000060961 | Slc4a4 | 1 | 1 | 1 | 1 | 1 | 0 | 0 | 0 | 0 | 0 |
| 61 | ENSMUSG00000020098 | Pcbd1 | 1 | 1 | 1 | 0 | 0 | 0 | 0 | 0 | 0 | 1 |
| 62 | ENSMUSG00000020914 | Top2a | 1 | 1 | 1 | 1 | 0 | 0 | 0 | 0 | 0 | 0 |
| 63 | ENSMUSG00000022336 | Eif3e | 1 | 1 | 1 | 1 | 0 | 0 | 0 | 0 | 0 | 0 |
| 64 | ENSMUSG00000063524 | Eno1 | 1 | 1 | 1 | 0 | 1 | 0 | 0 | 0 | 0 | 0 |
| 65 | ENSMUSG00000018362 | Kpna2 | 1 | 0 | 0 | 1 | 1 | 0 | 0 | 0 | 0 | 0 |
| 66 | ENSMUSG00000022881 | Rfc4 | 1 | 1 | 0 | 0 | 0 | 0 | 0 | 0 | 1 | 0 |
| 67 | ENSMUSG00000022962 | Gart | 1 | 1 | 1 | 0 | 0 | 0 | 0 | 0 | 0 | 0 |
| 68 | ENSMUSG00000027030 | Stk39 | 1 | 1 | 1 | 0 | 0 | 0 | 0 | 0 | 0 | 0 |
| 69 | ENSMUSG00000028970 | Abcb1b | 1 | 1 | 0 | 1 | 0 | 0 | 0 | 0 | 0 | 0 |
| 70 | ENSMUSG00000031278 | Acsl4 | 1 | 0 | 0 | 1 | 1 | 0 | 0 | 0 | 0 | 0 |
| 71 | ENSMUSG00000031928 | Mre11a | 1 | 1 | 1 | 0 | 0 | 0 | 0 | 0 | 0 | 0 |
| 72 | ENSMUSG00000054717 | Hmgb2 | 1 | 1 | 1 | 0 | 0 | 0 | 0 | 0 | 0 | 0 |
| 73 | ENSMUSG00000056209 | Npm3 | 1 | 1 | 0 | 1 | 0 | 0 | 0 | 0 | 0 | 0 |
| 74 | ENSMUSG00000001323 | Srr | 1 | 0 | 1 | 0 | 0 | 0 | 0 | 0 | 0 | 0 |
| 75 | ENSMUSG00000002984 | Tomm40 | 1 | 0 | 0 | 0 | 1 | 0 | 0 | 0 | 0 | 0 |
| 76 | ENSMUSG00000020649 | Rrm2 | 1 | 1 | 0 | 0 | 0 | 0 | 0 | 0 | 0 | 0 |
| 77 | ENSMUSG00000022234 | Cct5 | 1 | 0 | 1 | 0 | 0 | 0 | 0 | 0 | 0 | 0 |
| 78 | ENSMUSG00000026915 | Strbp | 1 | 1 | 0 | 0 | 0 | 0 | 0 | 0 | 0 | 0 |
| 79 | ENSMUSG00000027597 | Ahcy | 1 | 1 | 0 | 0 | 0 | 0 | 0 | 0 | 0 | 0 |
| 80 | ENSMUSG00000030662 | Ipo5 | 1 | 0 | 1 | 0 | 0 | 0 | 0 | 0 | 0 | 0 |
| 81 | ENSMUSG00000030978 | Rrm1 | 1 | 1 | 0 | 0 | 0 | 0 | 0 | 0 | 0 | 0 |
| 82 | ENSMUSG00000032481 | Smarcc1 | 1 | 1 | 0 | 0 | 0 | 0 | 0 | 0 | 0 | 0 |
| 83 | ENSMUSG00000046364 | Rpl27a | 1 | 1 | 0 | 0 | 0 | 0 | 0 | 0 | 0 | 0 |
| 84 | ENSMUSG00000056536 | Pign | 1 | 0 | 0 | 1 | 0 | 0 | 0 | 0 | 0 | 0 |
| 85 | ENSMUSG00000028010 | Gar1 | 0 | 0 | 0 | 0 | 0 | 0 | 0 | 0 | 0 | 0 |
| 86 | ENSMUSG00000030470 | Csrp3 | 0 | 0 | 0 | 0 | 0 | 0 | 0 | 0 | 0 | 0 |
| 87 | ENSMUSG00000053801 | Grwd1 | 0 | 0 | 0 | 0 | 0 | 0 | 0 | 0 | 0 | 0 |
| 88 | ENSMUSG00000032249 | Anp32a | 3 | 3 | 0 | 0 | 0 | 0 | 0 | 0 | 0 | 0 |
| 89 | ENSMUSG00000018102 | Hist1h2bc | 2 | 1 | 3 | 0 | 2 | 0 | 0 | 0 | 0 | 1 |
| 90 | ENSMUSG00000023927 | Satb1 | 1 | 1 | 0 | 0 | 0 | 0 | 0 | 0 | 0 | 0 |
