# Supplementary material for: c-Myc targeted regulators of cell metabolism in a transgenic mouse model of papillary lung adenocarcinoma
Source: Oncotarget. 2016 Sep 1;7(40):65514–39. doi: 10.18632/oncotarget.11804 (PMC5323172; doi:10.18632/oncotarget.11804)
Supplement: Supplementary file 4 [file oncotarget-07-65514-s004.docx]

**Supplementary Table S3: Master regulatory gene networks**

| **Master molecule (Entrez Gene ID)** | **Master molecule name** | **Maximal radius** | **Reachable total** | **Score** | **FDR** | **Z-Score** | **Ranks sum** | **Number of DEGs in PLAC** | **Gene in the network** | **Number of network elements found** | **Network element names** |
| --- | --- | --- | --- | --- | --- | --- | --- | --- | --- | --- | --- |
| 15382 | hnrpa1 | 4 | 2575 | 0,324749 | 0,035 | 1,874544 | 81 | 30 | abcb1b, AC147142.1, apex1, aqp4, arg1, eg433182, eg545091, eif3s6, eno1, gapdh, gpi1, hk2, hmgb2, hnrpa1, kpna2, ldha, lmnb1, mre11a, ncl, npm1, pcbd1, rrm1, rrs1, slc19a1, smarcc1, srr, timm8a1, tk1, xrcc5, xrcc6 | 30 | abl1, agt, ang1, atm, brca1, casp3, csnk2a1, dyrk1b, e2f1, eif2ak2, eif4g1, hdac9, hmgb1, igfbp3, il6, lmna, mapk8, nfkb1, nfkbia, pcna, rps27a, setd2, smad4, sp1, src, srebf1, stam2, tnf, trp53, wrn |
| 11792 | apex1 | 4 | 3306 | 0,444033 | 0,05 | 1,488 | 73 | 34 | abcb1b, AC147142.1, apex1, aqp4, arg1, eg433182, eg545091, eif3s6, eno1, fbl, gapdh, gpi1, hk2, hmgb2, hnrpa1, kpna2, ldha, lmnb1, mre11a, ncl, npm1, pcbd1, rfc4, rrm1, rrs1, slc19a1, slc4a4, smarcc1, srr, stk39, tk1, tomm40, xrcc5, xrcc6 | 36 | akt1, apaf1, apc, apoe, app, atm, bcl2, brca1, casp3, cbs, csnk2a1, csnk2a2, dhfr, dyrk1b, e2f1, eif4g1, golga3, hdac9, hdh, igfbp3, lmna, mdm2, nfkb1, nfkbia, pcna, prkaca, rad17, rb1, setd2, smad4, sp1, srebf1, tnf, trp53, wnk1, wrn |
| 21991 | tpi1 | 4 | 1568 | 0,256466 | 0,031 | 2,32861 | 70 | 19 | AC147142.1, abcb1b, apex1, eg545091, eif3s6, gapdh, hk2, hnrpa1, kpna2, lmnb1, mre11a, ncl, npm1, slc4a4, srr, tk1, tpi1, xrcc5, xrcc6 | 18 | abcc8, atm, bax, brca1, casp3, eif4g1, golga3, kcnj11, lmna, mdm2, myc, nfkb1, nfkbia, parp1, prkaca, srebf1, tnf, trp53 |
| 20509 | slc19a1 | 4 | 2794 | 0,389163 | 0,028 | 1,994855 | 58 | 31 | abcb1b, AC147142.1, apex1, aqp4, arg1, eg433182, eg545091, eno1, gapdh, gpi1, hmgb2, hnrpa1, kpna2, ldha, mre11a, ncl, npm1, rfc4, rrm1, rrm2, rrs1, shmt1, slc19a1, slc4a4, smarcc1, srr, timm8a1, tk1, tomm40, xrcc5, xrcc6 | 29 | agt, apc, chka, csnk2a1, csnk2a2, dhfr, e2f1, egfr, ep300, igfbp3, mapk3, mknk1, mtx1, myc, nfkbia, pcna, prkaca, rad17, rpa1, rps27a, setd2, smad4, sp1, src, stam2, thf, tnf, trp53, wrn |
| 17975 | ncl | 4 | 3545 | 0,633373 | 0,005 | 1,351353 | 55 | 37 | abcb1b, AC147142.1, apex1, aqp4, arg1, eg433182, eg545091, eif3s6, eno1, fbl, gapdh, gpi1, hk2, hmgb2, hnrpa1, kpna2, ldha, lmnb1, mre11a, ncl, npm1, pcbd1, rfc4, rrm1, rrm2, rrs1, slc19a1, slc4a4, smarcc1, srr, stk39, timm8a1, tk1, tomm40, tpi1, xrcc5, xrcc6 | 41 | akt1, apoe, atm, brca1, casp3, cbs, csnk2a1, csnk2a2, dhfr, dyrk1b, e2f1, eif4g1, gadd45a, hdac9, hif1a, hsp90aa1, igfbp3, kcnj11, lmna, mdk, myb, nfkb1, nfkbia, nos2, parp1, pcna, prkaca, rad17, rpa1, rps27a, setd2, smad4, sp1, src, srebf1, stam2, tnf, trp53, wnk1, wrn, yy1 |
| 100046628 | npm1 | 4 | 3464 | 0,617984 | 0,01 | 1,410637 | 50 | 37 | abcb1b, AC147142.1, apex1, aqp4, arg1, eg433182, eg545091, eif3s6, eno1, fbl, gapdh, gpi1, hk2, hmgb2, hnrpa1, kpna2, ldha, lmnb1, mre11a, ncl, pcbd1, rfc4, rrm1, rrm2, rrs1, slc19a1, slc4a4, smarcc1, srr, stk39, timm8a1, tk1, tomm40, tpi1, xrcc5, xrcc6 | 38 | agt, akt1, ar, atm, brca1, cbs, cdkn2b, chek2, csnk2a1, csnk2a2, dhfr, dyrk1b, e2f1, eif2ak2, eif4e, eif4g1, fos, hdac9, htr, igfbp3, kcnj11, lmna, mdm2, myc, nfkbia, pcna, prkaca, rad17, rps27a, setd2, smad4, sp1, srebf1, stam2, stat3, tnf, trp53, wnk1 |
| 14433 | gapdh | 4 | 3443 | 0,588738 | 0,015 | 1,528706 | 49 | 35 | abcb1b, AC147142.1, apex1, aqp4, arg1, eg433182, eg545091, eif3s6, eno1, fbl, gapdh, gpi1, hk2, hmgb2, hnrpa1, kpna2, ldha, lmnb1, mre11a, ncl, npm1, pcbd1, rrm1, rrs1, slc19a1, slc4a4, smarcc1, srr, stk39, timm8a1, tk1, tomm40, tpi1, xrcc5, xrcc6 | 41 | adra1d, agt, akt1, atxn1, brca1, c5ar1, casp3, cbs, chek2, csnk2a1, dhfr, dyrk1b, e2f1, egf, eif4g1, furin, hdh, hmgb1, igfbp3, itpr1, kcnj11, lmna, map2k3, mapk8, myc, nfkbia, pcna, plg, plk3, pml, prkaca, rps27a, setd2, smad4, sp1, srebf1, stam2, stat3, tnf, trp53, wnk1 |

| **Integrated network of 7 master regulators** | **Number of hits (DEGs) found** | **Hit names** | **Number of network elements found** | **Network element names** |
| --- | --- | --- | --- | --- |
| apex1, gapdh, hnrpa1, ncl, npm1, slc19a1, tpi1 | 38 | abcb1b, AC147142.1, apex1, aqp4, arg1, eg433182, eg545091, eif3s6, eno1, fbl, gapdh, gpi1, hk2, hmgb2, hnrpa1, kpna2, ldha, lmnb1, mre11a, ncl, npm1, pcbd1, rfc4, rrm1, rrm2, rrs1, shmt1, slc19a1, slc4a4, smarcc1, srr, stk39, timm8a1, tk1, tomm40, tpi1, xrcc5, xrcc6 | 83 | abcc8, abl1, adra1d, agt, akt1, ang1, apaf1, apc, apoe, app, ar, atm, atxn1, bax, bcl2, brca1, c5ar1, casp3, cbs, cdkn2b, chek2, chka, csnk2a1, csnk2a2, dhfr, dyrk1b, e2f1, egf, egfr, eif2ak2, eif4e, eif4g1, ep300, fos, furin, gadd45a, golga3, hdac9, hdh, hif1a, hmgb1, hsp90aa1, htr, igfbp3, il6, itpr1, kcnj11, lmna, map2k3, mapk3, mapk8, mdk, mdm2, mknk1, mtx1, myb, myc, nfkb1, nfkbia, nos2, parp1, pcna, plg, plk3, pml, prkaca, rad17, rb1, rpa1, rps27a, setd2, smad4, sp1, src, srebf1, stam2, stat3, thf, tnf, trp53, wnk1, wrn, yy1 |

**Note: Hits define up-regulated genes in PLACs and network elements are connecting genes provided by the GeneWays database**
