# Supplementary material for: c-Myc targeted regulators of cell metabolism in a transgenic mouse model of papillary lung adenocarcinoma
Source: Oncotarget. 2016 Sep 1;7(40):65514–39. doi: 10.18632/oncotarget.11804 (PMC5323172; doi:10.18632/oncotarget.11804)
Supplement: Supplementary file 5 [file oncotarget-07-65514-s005.docx]

**Supplementary Table S5: Co-occupancy of c-Myc and TFDP1 transcription factor binding sites in PLAC regulated genes**

| **ID** | **Name** | **TF-Binding Sites (V$E2F_Q4_01)** | **TF-Binding Sites (V$EBOX_Q6_01)** | **Score** |
| --- | --- | --- | --- | --- |
| ENSMUSG00000025007 | Aldh18a1 | 2 | 2 | 3,551518202 |
| ENSMUSG00000046434 | Hnrnpa1 | 2 | 2 | 3,487312317 |
| ENSMUSG00000046865 | Fbl | 2 | 2 | 3,468307018 |
| ENSMUSG00000026234 | Ncl | 2 | 2 | 3,406076908 |
| ENSMUSG00000057278 | Snrpg | 2 | 2 | 3,398159504 |
| ENSMUSG00000000628 | Hk2 | 2 | 2 | 3,362401724 |
| ENSMUSG00000061024 | Rrs1 | 2 | 2 | 3,235368252 |
| ENSMUSG00000025747 | Tyms | 2 | 2 | 3,226028919 |
| ENSMUSG00000035960 | Apex1 | 2 | 2 | 3,10471344 |
| ENSMUSG00000024590 | Lmnb1 | 2 | 2 | 3,029479504 |
| ENSMUSG00000032518 | Rpsa | 2 | 2 | 2,930884838 |
| ENSMUSG00000006442 | Srm | 2 | 2 | 2,892192125 |
| ENSMUSG00000031388 | Naa10 | 2 | 2 | 2,843388557 |
| ENSMUSG00000032892 | Rangrf | 1 | 2 | 2,755572557 |
| ENSMUSG00000027405 | Nop56 | 2 | 2 | 2,732396841 |
| ENSMUSG00000037805 | Rpl10a | 2 | 2 | 2,684580564 |
| ENSMUSG00000037012 | Hk1 | 1 | 2 | 2,663954258 |
| ENSMUSG00000054717 | Hmgb2 | 2 | 1 | 2,652505398 |
| ENSMUSG00000025153 | Fasn | 2 | 2 | 2,639615059 |
| ENSMUSG00000057113 | Npm1 | 2 | 1 | 2,637239933 |
| ENSMUSG00000020547 | Bzw2 | 2 | 2 | 2,625489235 |
| ENSMUSG00000029642 | Polr1d | 2 | 2 | 2,545707464 |
| ENSMUSG00000022336 | Eif3e | 2 | 1 | 2,527485371 |
| ENSMUSG00000022962 | Gart | 2 | 1 | 2,479291201 |
| ENSMUSG00000048007 | Timm8a1 | 1 | 2 | 2,445271969 |
| ENSMUSG00000027076 | Timm10 | 2 | 2 | 2,439922094 |
| ENSMUSG00000074129 | Rpl13a | 2 | 1 | 2,418294907 |
| ENSMUSG00000079435 | Rpl36a | 1 | 2 | 2,413651943 |
| ENSMUSG00000031928 | Mre11a | 2 | 1 | 2,397827625 |
| ENSMUSG00000020534 | Shmt1 | 2 | 2 | 2,38734436 |
| ENSMUSG00000062867 | Impdh2 | 2 | 2 | 2,379849911 |
| ENSMUSG00000060961 | Slc4a4 | 2 | 1 | 2,337198496 |
| ENSMUSG00000020098 | Pcbd1 | 2 | 1 | 2,266558647 |
| ENSMUSG00000021474 | Sfxn1 | 2 | 2 | 2,20563817 |
| ENSMUSG00000002319 | Ipo4 | 2 | 1 | 2,202700853 |
| ENSMUSG00000026187 | Xrcc5 | 2 | 2 | 2,157642841 |
| ENSMUSG00000021556 | Golm1 | 2 | 1 | 2,129848719 |
| ENSMUSG00000021733 | Slc4a7 | 2 | 2 | 2,127337933 |
| ENSMUSG00000024411 | Aqp4 | 1 | 2 | 2,126461506 |
| ENSMUSG00000026915 | Strbp | 2 | 1 | 2,108776569 |
| ENSMUSG00000036427 | Gpi1 | 2 | 2 | 2,096203804 |
| ENSMUSG00000025574 | Tk1 | 2 | 2 | 2,049503326 |
| ENSMUSG00000057666 | Gapdh | 2 | 2 | 2,02472353 |
| ENSMUSG00000031754 | Nudt21 | 2 | 1 | 1,998370767 |
| ENSMUSG00000024785 | Rcl1 | 2 | 2 | 1,966310978 |
| ENSMUSG00000003355 | Fkbp11 | 1 | 2 | 1,91755867 |
| ENSMUSG00000011257 | Pabpc4 | 0 | 2 | 1,893309474 |
| ENSMUSG00000019987 | Arg1 | 2 | 2 | 1,822490811 |
| ENSMUSG00000022899 | Slc15a2 | 1 | 1 | 1,786004066 |
| ENSMUSG00000044927 | H1fx | 2 | 1 | 1,716569781 |
| ENSMUSG00000039640 | Mrpl12 | 2 | 0 | 1,699995518 |
| ENSMUSG00000020914 | Top2a | 2 | 1 | 1,683889747 |
| ENSMUSG00000027374 | Mrps5 | 2 | 1 | 1,671443343 |
| ENSMUSG00000020649 | Rrm2 | 2 | 0 | 1,66488409 |
| ENSMUSG00000029388 | Eif2b1 | 2 | 0 | 1,652130961 |
| ENSMUSG00000032481 | Smarcc1 | 2 | 0 | 1,647000432 |
| ENSMUSG00000022881 | Rfc4 | 2 | 1 | 1,646877408 |
| ENSMUSG00000001436 | Slc19a1 | 2 | 2 | 1,642433405 |
| ENSMUSG00000056209 | Npm3 | 2 | 0 | 1,611049652 |
| ENSMUSG00000030978 | Rrm1 | 2 | 0 | 1,598926783 |
| ENSMUSG00000046364 | Rpl27a | 2 | 0 | 1,596625686 |
| ENSMUSG00000026377 | Mki67ip | 2 | 0 | 1,500976563 |
| ENSMUSG00000028970 | Abcb1b | 2 | 0 | 1,458773613 |
| ENSMUSG00000026558 | Uck2 | 2 | 0 | 1,337330937 |
| ENSMUSG00000027597 | Ahcy | 2 | 0 | 1,293938994 |
| ENSMUSG00000023456 | Tpi1 | 1 | 2 | 1,154527068 |
| ENSMUSG00000063229 | Ldha | 1 | 1 | 1,07157743 |
| ENSMUSG00000061838 | Suclg2 | 1 | 2 | 1,013790727 |
| ENSMUSG00000024640 | Psat1 | 1 | 1 | 0,98296982 |
| ENSMUSG00000004100 | Ppan | 2 | 1 | 0,978980064 |
| ENSMUSG00000037722 | Gnpnat1 | 0 | 1 | 0,971759975 |
| ENSMUSG00000063524 | Eno1 | 0 | 1 | 0,94936502 |
| ENSMUSG00000027030 | Stk39 | 0 | 1 | 0,947143972 |
| ENSMUSG00000001323 | Srr | 0 | 0 | 0 |
| ENSMUSG00000002984 | Tomm40 | 0 | 0 | 0 |
| ENSMUSG00000018362 | Kpna2 | 0 | 0 | 0 |
| ENSMUSG00000022234 | Cct5 | 0 | 0 | 0 |
| ENSMUSG00000022471 | Xrcc6 | 0 | 0 | 0 |
| ENSMUSG00000025001 | Hells | 0 | 0 | 0 |
| ENSMUSG00000026020 | Nop58 | 0 | 0 | 0 |
| ENSMUSG00000028010 | Gar1 | 0 | 0 | 0 |
| ENSMUSG00000030470 | Csrp3 | 0 | 0 | 0 |
| ENSMUSG00000030662 | Ipo5 | 0 | 0 | 0 |
| ENSMUSG00000031278 | Acsl4 | 0 | 0 | 0 |
| ENSMUSG00000053801 | Grwd1 | 0 | 0 | 0 |
| ENSMUSG00000056536 | Pign | 0 | 0 | 0 |
| ENSMUSG00000065087 | Snord22 | 0 | 0 | 0 |
