# Supplementary material for: c-Myc targeted regulators of cell metabolism in a transgenic mouse model of papillary lung adenocarcinoma
Source: Oncotarget. 2016 Sep 1;7(40):65514–39. doi: 10.18632/oncotarget.11804 (PMC5323172; doi:10.18632/oncotarget.11804)
Supplement: Supplementary file 7 [file oncotarget-07-65514-s007.docx]

**Supplementary Table S8: Comparison EMSA data of the current study with published Ref-seq/Chip-seq data**

| **EMSA studied genes, see Figure 5** | **Sabò et al., 2014 [18]** | | | **ChIP-seq data**  **Walz et al, 2014 [19]** | | |
| --- | --- | --- | --- | --- | --- | --- |
|  | **ChIP-seq signal** | **Fold P/C** | **Fold T/C** | **T cells** | **MEFs** | **Pancreas cells** |
| **Shmt1** | 9,52 | 3,96 | 4,39 | + | + | + |
| **Hk1** | 7,61 | 0,72 | 0,41 | + | - | + |
| **Gapdh** | 9,25 | 1,44 | 2,41 | + | + | + |
| **Tpi** | 8,38 | 3,34 | 4,04 | - | + | - |
| **Gart** | 10,24 | 2,37 | 2,44 | + | + | + |
| **Impdh2** | 9,14 | 2,36 | 3,00 | + | + | + |
| **Uck2** | 11,20 | 7,29 | 10,75 | - | + | + |
| **Apex1** | 9,41 | 2,21 | 2,75 | - | + | - |
| **Rcl1** | 10,13 | 2,79 | 3,13 | + | + | + |
| **Pabpc4** | 9,35 | 2,11 | 2,58 | + | + | + |
| **Gar1** | 10,67 | 2,37 | 2,81 | + | + | + |
| **Ppan** | 11,20 | 2,15 | 3,19 | + | + | + |
| **Rpl27a** | 10,27 | 1,56 | 1,42 | + | + | + |
| **Bzw2** | 10,47 | 2,93 | 3,43 | + | + | + |
| **Eif3e** | 10,26 | 1,54 | 1,33 | + | + | + |
| **Cct5** | 10,30 | 2,03 | 1,87 | + | + | + |
| **Abcb1b** | 8,76 | 1,18 | 6,11 | - | - | + |
| **Smarcc1** | 9,28 | 2,04 | 1,96 | + | + | + |
| **Nop56** | 11,63 | 1,97 | 2,91 | + | + | + |
| **Ncl** | 11,90 | 2,50 | 2,91 | - | - | - |
| **Npm1** | 11,10 | 3,34 | 3,65 | - | - | - |
| **Npm3** | 8,95 | 2,27 | 3,05 | + | + | + |
| **Hnrnpa1** | 11,27 | 1,68 | 1,67 | - | - | + |
| **Fbl** | 10,63 | 2,42 | 2,55 | + | + | + |
| **Nop58** | 10,55 | 2,48 | 3,22 | + | + | + |
| **Fkbp11** | 8,69 | 3,18 | 4,46 | - | + | - |
| **Aldh18A1** | 10,02 | 1,85 | 1,96 | + | + | + |
| **Rpsa** | 9,82 | 1,69 | 1,83 | + | + | + |
| **Timm10** | 9,17 | 1,60 | 1,96 | + | + | + |
| **Timm8a** | 1,93 | 0,91 | 1,73 | + | + | + |
| **Slc19a1** | 10,83 | 3,46 | 6,13 | - | - | + |
| **Rangrf** | 8,54 | n.a. | n.a. | + | - | + |
| **Csrp3** | 0,00 | n.a. | n.a. | - | - | - |
|  | % binding > 2 | % > 1.5 | % > 1.5 | % + | % + | % + |
