# Supplementary material for: c-Myc targeted regulators of cell metabolism in a transgenic mouse model of papillary lung adenocarcinoma
Source: Oncotarget. 2016 Sep 1;7(40):65514–39. doi: 10.18632/oncotarget.11804 (PMC5323172; doi:10.18632/oncotarget.11804)
Supplement: Supplementary file 8 [file oncotarget-07-65514-s008.docx]

**Supplementary Table S9: Hazard ratios for individual PLAC regulated genes**

| **Up-regulated** | **Gene** | **HR** | **P Value** | **N** |
| --- | --- | --- | --- | --- |
|  | **Arg1** | 2.4 | 3,2E-13 | 720 |
|  | **Fasn** | 1.79 | 0,0000011 | 720 |
|  | **Srm** | 2.35 | 2,3E-11 | 720 |
|  | **Shmt1** | 1.43 | 0,0023 | 720 |
|  | **Hk2** | 1.59 | 0,00019 | 720 |
|  | **Hk1** | 0.93 | 0,55 | 720 |
|  | **Gapdh** | 3.45 | 1E-17 | 720 |
|  | **Tpi** | 2.17 | 2,7E-10 | 720 |
|  | **Tk1** | 2.71 | 4,3E-15 | 673 |
|  | **Impdh2** | 0.71 | 0,0044 | 720 |
|  | **Uck2** | 2.22 | 1,7E-10 | 673 |
|  | **Top2a** | 1.81 | 8,6E-07 | 720 |
|  | **Hmgb2** | 0.63 | 0,000089 | 720 |
|  | **Apex1** | 0.79 | 0,048 | 720 |
|  | **Rrm2** | 1.39 | 0,0062 | 720 |
|  | **Smarcc1** | 1.85 | 3,1E-07 | 720 |
|  | **H1fx** | 1.71 | 0,0000076 | 720 |
|  | **Ncl** | 1.00 | 0,99 | 720 |
|  | **Npm1** | 1.35 | 0,014 | 720 |
|  | **Npm3** | 2.09 | 7,2E-10 | 720 |
|  | **Hnrpa1** | 0.75 | 0,015 | 720 |
|  | **Rcl1** | 0.74 | 0,014 | 673 |
|  | **Rpsa** | 1.4 | 0,005 | 720 |
|  | **Slc19a1** | 2.07 | 1,3E-09 | 720 |
|  | **Slc4a4** | 0.56 | 0,0000031 | 673 |
|  | **Kpna2** | 1.76 | 0,0000026 | 720 |
|  | **Nop56** | 2.21 | 1,7E-09 | 720 |
|  | **Nop58** | 0.94 | 0,63 | 673 |
|  | **Nifk** | 1.78 | 0,0000021 | 673 |
|  | **Aldh18A1** | 1.78 | 0,0000015 | 673 |
|  | **Gpi1** | 1.49 | 0,0018 | 720 |
|  | **Strbp** | 0.59 | 0,000032 | 673 |
|  | **Eno1** | 1.96 | 0,0000002 | 673 |
|  | **Ldha** | 2.02 | 9E-09 | 720 |
|  | **Ppan** | 1.12 | 0,34 | 720 |
|  | **Gar1** | 0.91 | 0,43 | 720 |
|  | **Mre11a** | 0.72 | 0,0073 | 673 |
|  | **Acsl4** | 0.82 | 0,1 | 720 |
|  | **Xrcc5** | 0.78 | 0,034 | 720 |
|  | **Xrcc6** | 0.87 | 0,24 | 720 |
|  | **Fbl** | 1.26 | 0,054 | 720 |
|  | **Gart** | 1.1 | 0,4 | 720 |
|  | **Timm10** | 1.22 | 0,098 | 720 |
|  | **Tomm40** | 2.08 | 1,3E-09 | 720 |
|  | **Timm8a** | 0.87 | 0,23 | 720 |
|  | **Suclg2** | 0.51 | 1,2E-08 | 720 |
|  | **Gnpnat1** | 0.75 | 0,019 | 673 |
|  | **Pign** | 0.53 | 4,5E-07 | 673 |
|  | **Hells** | 1.32 | 0,025 | 673 |
|  | **Tyms** | 1.87 | 0,0000006 | 673 |
|  | **Ahcy** | 1.16 | 0,2 | 720 |
|  | **Naa10** | - | - | - |
|  | **Rrm1** | 0.98 | 0,87 | 720 |
|  | **Rfc4** | 1.78 | 0,0000022 | 720 |
|  | **Lmnb1** | 2.02 | 5,7E-09 | 720 |
|  | **Polr1d** | 1.28 | 0,049 | 673 |
|  | **Rrs1** | 1.13 | 0,3 | 720 |
|  | **Snrpg** | 0.92 | 0,46 | 673 |
|  | **Snord22** | - | - | - |
|  | **Nudt21** | 0.63 | 0,00015 | 673 |
|  | **Pabpc4** | 0.84 | 0,13 | 720 |
|  | **Grwd1** | 1.49 | 0,00071 | 720 |
|  | **Psat1** | 1.56 | 0,0003 | 720 |
|  | **Rpl10a** | 1.65 | 0,0000065 | 673 |
|  | **Rpl13a** | 1.95 | 3,8E-08 | 720 |
|  | **Rpl27a** | 1.92 | 2,7E-09 | 720 |
|  | **Bzw2** | 1.17 | 0,19 | 720 |
|  | **Eif3e** | 0.6 | 0,000015 | 720 |
|  | **Cct5** | 1.91 | 4,2E-07 | 673 |
|  | **Fkbp11** | 1.72 | 0,0000092 | 720 |
|  | **Srr** | 1.01 | 0,96 | 673 |
|  | **Pcbd1** | 1.43 | 0,0032 | 720 |
|  | **Mrps5** | 1.55 | 0,00036 | 673 |
|  | **Mrpl12** | 2.2 | 1,3E-10 | 720 |
|  | **Rpl36a** | 2.07 | 9,2E-09 | 720 |
|  | **Eif2b1** | 0.81 | 0,077 | 720 |
|  | **Golm1** | 0.84 | 0,14 | 720 |
|  | **Sfxn1** | 1.35 | 0,017 | 673 |
|  | **Slc15a2** | 0.56 | 0,0000015 | 720 |
|  | **Slc4a7** | 0.69 | 0,0021 | 720 |
|  | **Aqp4** | 0.7 | 0,0034 | 673 |
|  | **Rangrf** | 1.51 | 0,001 | 673 |
|  | **Ipo5** | 0.64 | 0,00018 | 720 |
|  | **Ipo4** | 2.21 | 9,6E-11 | 720 |
|  | **Csrp3** | 1.71 | 0,0000071 | 720 |
|  | **Abcb1b** | 0.6 | 0,0000016 | 720 |
|  | **Stk39** | 0.64 | 0,00013 | 720 |
| **Down-regulated** | **Gene** | **HR** | **P Value** | **N** |
|  | **Satb1** | 0.48 | 8,6E-09 | 673 |
|  | **Anp32a** | 0.51 | 2,6E-08 | 673 |
|  | **Hist1h2bc** | 1.43 | 0,0026 | 673 |
